# Bivalent ligands promote endosomal trafficking of the dopamine D3 receptor-neurotensin receptor 1 heterodimer

**DOI:** 10.1038/s42003-021-02574-4

**Published:** 2021-09-10

**Authors:** Julian Budzinski, Simone Maschauer, Hiroyuki Kobayashi, Pierre Couvineau, Hannah Vogt, Peter Gmeiner, Anna Roggenhofer, Olaf Prante, Michel Bouvier, Dorothee Weikert

**Affiliations:** 1 Department of Chemistry and Pharmacy, Medicinal Chemistry, Friedrich-Alexander-Universität Erlangen-Nürnberg, Erlangen, Germany; 2 Department of Nuclear Medicine, Molecular Imaging and Radiochemistry, Friedrich-Alexander-Universität Erlangen-Nürnberg, Erlangen, Germany; 3 Department of Biochemistry and Molecular Medicine, Institute for Research in Immunology and Cancer, Université de Montréal, Montreal, QC Canada

**Keywords:** Receptor pharmacology, Receptor pharmacology

## Abstract

Bivalent ligands are composed of two pharmacophores connected by a spacer of variable size. These ligands are able to simultaneously recognize two binding sites, for example in a G protein-coupled receptor heterodimer, resulting in enhanced binding affinity. Taking advantage of previously described heterobivalent dopamine-neurotensin receptor ligands, we demonstrate specific interactions between dopamine D3 (D_3_R) and neurotensin receptor 1 (NTSR1), two receptors with expression in overlapping brain areas that are associated with neuropsychiatric diseases and addiction. Bivalent ligand binding to D_3_R-NTSR1 dimers results in picomolar binding affinity and high selectivity compared to the binding to monomeric receptors. Specificity of the ligands for the D_3_R-NTSR1 receptor pair over D_2_R-NTSR1 dimers can be achieved by a careful choice of the linker length. Bivalent ligands enhance and stabilize the receptor-receptor interaction leading to NTSR1-controlled internalization of D_3_R into endosomes via recruitment of β-arrestin, highlighting a potential mechanism for dimer-specific receptor trafficking and signalling.

## Abstract (web summary)

Budzinski et al use bivalent ligands, BRET assays and radioligand competition to demonstrate a specific interaction between two receptors associated with neuropsychiatric diseases and addiction, dopamine D3 (D_3_R) and neurotensin receptor 1. They show that the bivalent ligands promote endosomal trafficking of D_3_R, suggesting a potential role for dimerization in vivo.

## Introduction

**Fig. 1:**
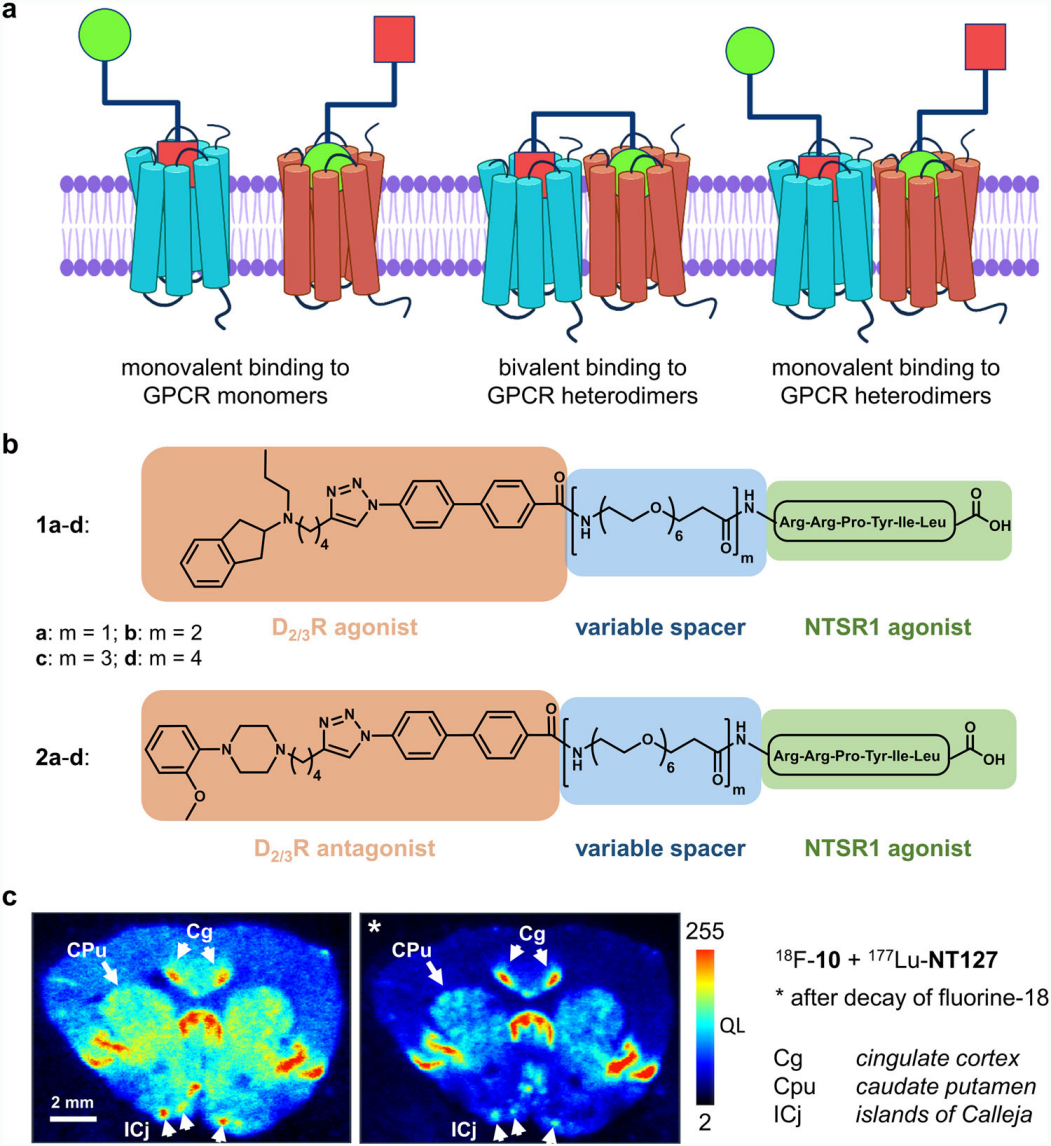
Bivalent ligands targeting heterodimers and distribution of D_3_R and NTSR1 in the rat CNS. **a** Schematic representation of binding modes of heterobivalent ligands at dimeric or monomeric GPCRs, created with biorender.com. **b** Bivalent ligands used in the present study are composed of a dopamine receptor agonist (**1a**-**d**) or antagonist (**2a**-**d**), a linker of flexible size (*m* = 1-4 PEG-spacer units) and NT(8-13) as peptide NTSR1 agonist. **c** In vitro rat brain autoradiography with the D_3_R-selective radioligand ^18^F-**10**^39^ and the NTSR1-selective radioligand ^177^Lu-**NT127**^40^ visualises the expression of D_3_R and NTSR1. The first image in **c** is taken directly after labelling, the second autoradiography was taken 24 h later, after complete radioactive decay of ^18^F, showing only expression of NTSR1. Areas labelled with the white arrows indicate coexpression of D_3_R and NTSR1. The intensity is shown as quantum level (QL) as provided by the software Quantity One.

Specific interactions of class A G protein-coupled receptors (GPCRs), such as the formation of homo- or heterodimers or higher order oligomers, influence signalling properties, binding affinities, receptor trafficking or enable cross-talk^1–5^ and have inspired the development of bivalent ligands^6,7^. Following the leads of studies reporting ligands that selectively engage opioid receptor dimers^8^, medicinal chemists have aimed at developing bivalent ligands for different class A GPCRs^9^. Comprising two pharmacophores connected to each other by a spacer, bivalent ligands are able to simultaneously engage two distinct binding sites located in the two individual protomers of a dimer^9^ (Fig. 1a). As a result of the thermodynamic advantage over the monovalent binding to either receptor, they confer higher affinities and also selectivity towards their combined recognition elements^7^. Besides the undoubted value of bivalent ligands as pharmacological tools for the study of the mechanism and functional consequences of receptor-receptor interactions, targeting GPCR dimers represents a promising therapeutic approach^10^.

We have previously reported a series of bivalent ligands targeting heterodimers of dopamine D2 (D_2_R) and neurotensin receptors 1 (NTSR1)^11^. These ligands exhibit more than 1000-fold selectivity for cells coexpressing the two receptors over cells expressing only the D_2_R and moderate selectivity over monomeric NTSR1.

Dopamine D_3_ receptors (D_3_R) share 52% sequence identity (75% identity within the transmembrane regions) with D_2_R^12^ and have been reported to be involved in multiple neuropsychiatric diseases including Schizophrenia^13^, Parkinson’s Disease^14^, and more recently neuroinflammation^15^. Besides the expression in the nucleus accumbens and caudate putamen, D_3_R is highly expressed in the islands of Calleja^16,17^, a brain region involved in reward seeking behaviour^18^. Importantly, D_3_R is upregulated in the context of various drug addictions and targeting D_3_R with monovalent antagonists or partial agonists is seen as a promising avenue in the context of drug abuse treatment^19–22^.

NTSR1 is expressed in different areas of the central nervous system (CNS) including the hypothalamus, the basal forebrain and the limbic system^23^. Using immunohistochemical staining, high levels of NTSR1 have also been observed in the islands of Calleja^24^. The distribution of NTSR1 reflects its role in the modulation of numerous processes, including locomotion, memory and cognition^25^. Various attempts for the clinical development of NTSR1 agonists and antagonists as neuroleptics have been made, but efforts were hampered by undesired effects including hypotension, hypothermia and motor impairment^26^. Very recently, allosteric modulation and β-arrestin biased signalling of NTSR1 have been identified as novel and promising strategies in the field of addiction therapy^27,28^.

While NTSR1 is able to form homodimers^29^, it also interacts with different class A GPCRs like D_2_R and NTSR2, resulting in heterodimer-specific ligand binding, signalling and trafficking^30–34^. Interestingly, the dopamine and neurotensin neurotransmitter systems are interconnected in the CNS and known to modulate each other^35,36^. Besides direct influences of NTSR1 on D_2_R, negative modulation of the D_3_R by NTSR1 resulting in a reduced binding affinity of dopamine receptor agonists has been observed in transfected HEK293 cells^37^. Moreover, D_3_R and neurotensin mRNA show overlapping distribution in the rat *nucleus accumbens*^36^ and neurotensin has been shown to diminish 7-OH-DPAT affinity for D_3_R in limbic areas of the rat brain^38^. However, bivalent ligands targeting D_3_R-NTSR1 heterodimers have not yet been reported.

In this study, we investigate potential D_3_R-NTSR1 heterodimer formation and examine how bivalent ligands affect the pharmacology and the trafficking of the receptors. We use our previously described bivalent D_2_R-NTSR1 ligands **1a**-**d** and **2a**-**d** (Fig. 1b)^11^ because their dopaminergic pharmacophores are not selective among members of the D_2_-like receptor subfamily (D_2_R, D_3_R and D_4_R). Using a combination of ligand binding and bioluminescence resonance energy transfer (BRET) assays, we demonstrate that bivalent ligand binding at D_3_R-NTSR1 heteromeric complexes occurs at very low ligand concentration and fosters the interaction of the D_3_R and NTSR1 protomers. Interestingly, the ligands **1a** and **2a** comprising a short linker showed selective binding to D_3_R-NTSR1 heterodimers over D_2_R-NTSR1 heterodimers. In contrast to stimulation with monovalent ligands, bivalent engagement of D_3_R-NTSR1 dimers leads to internalisation of the heteromeric complex, revealing unique pharmacological properties of bivalent ligands. As ongoing research seeks to evaluate how upregulation, binding potential and function of the D_3_R are linked to substance abuse^20^, our findings regarding altered receptor trafficking and β-arrestin recruitment properties of the D_3_R may pave the way towards novel therapeutic approaches, in particular since D_3_R and NTSR1 show expression in overlapping brain regions, even if direct evidence for their in vivo dimerization and sub-cellular colocalization remains yet to be provided.

## Results

### D_3_R and NTSR1 expression shows substantial overlap within rat brain

To visualise the regional distribution of D_3_R and NTSR1 in the CNS, we performed in vitro rat brain autoradiography of striatal slices employing concomitant incubation with both the D_3_R- and NTSR1-selective radioligands ^18^F-**10**^39^ and ^177^Lu-**NT127**^40^, respectively. The D_3_R-selective radioligand ^18^F-**10** alone resulted in a labelling consistent with the known regional distribution of the D_3_R in the rat brain^41^, including caudate putamen (CPu), nucleus accumbens (NAc), cingulate cortex (Cg) and the islands of Calleja (ICj) (Supplementary Fig. 1a). Using the selective NTSR1 radioligand ^177^Lu-**NT127**, high levels of NTSR1-binding sites were found in the rhinal sulcus (SR), cingulate cortex (Cg), medial septum (MS) and the islands of Calleja (ICj) and low to moderate density of binding sites in the nucleus accumbens (Supplementary Fig. 1b), confirming the reported NTSR1 distribution in rat brain as determined by autoradiography with [^3^H]SR142948A and immunohistochemical staining of the NTSR1^24,42^. Coincubation of rat brain slices with both radioligands showed the sum of NTSR1 and D_3_R-binding sites, as depicted in Fig. 1c, with non-specific binding determined in the presence of neurotensin (1 µM, Supplementary Fig. 1d) or the D_3_R ligand BP897 (50 nM, Supplementary Fig. 1e). After the decay of fluorine-18 (Supplementary Fig. 1a*-e*), the resulting autoradiography of the remaining ^177^Lu-labelled binding sites revealed colocalization of NTSR1 and D_3_R receptors in the cingulate cortex (Cg), caudate putamen (Cpu) and the islands of Calleja (ICj) of rat brain striatal slices (Fig. 1c).

### Bivalent ligands bind to D_3_R-NTSR1 with high affinity

**Fig. 2:**
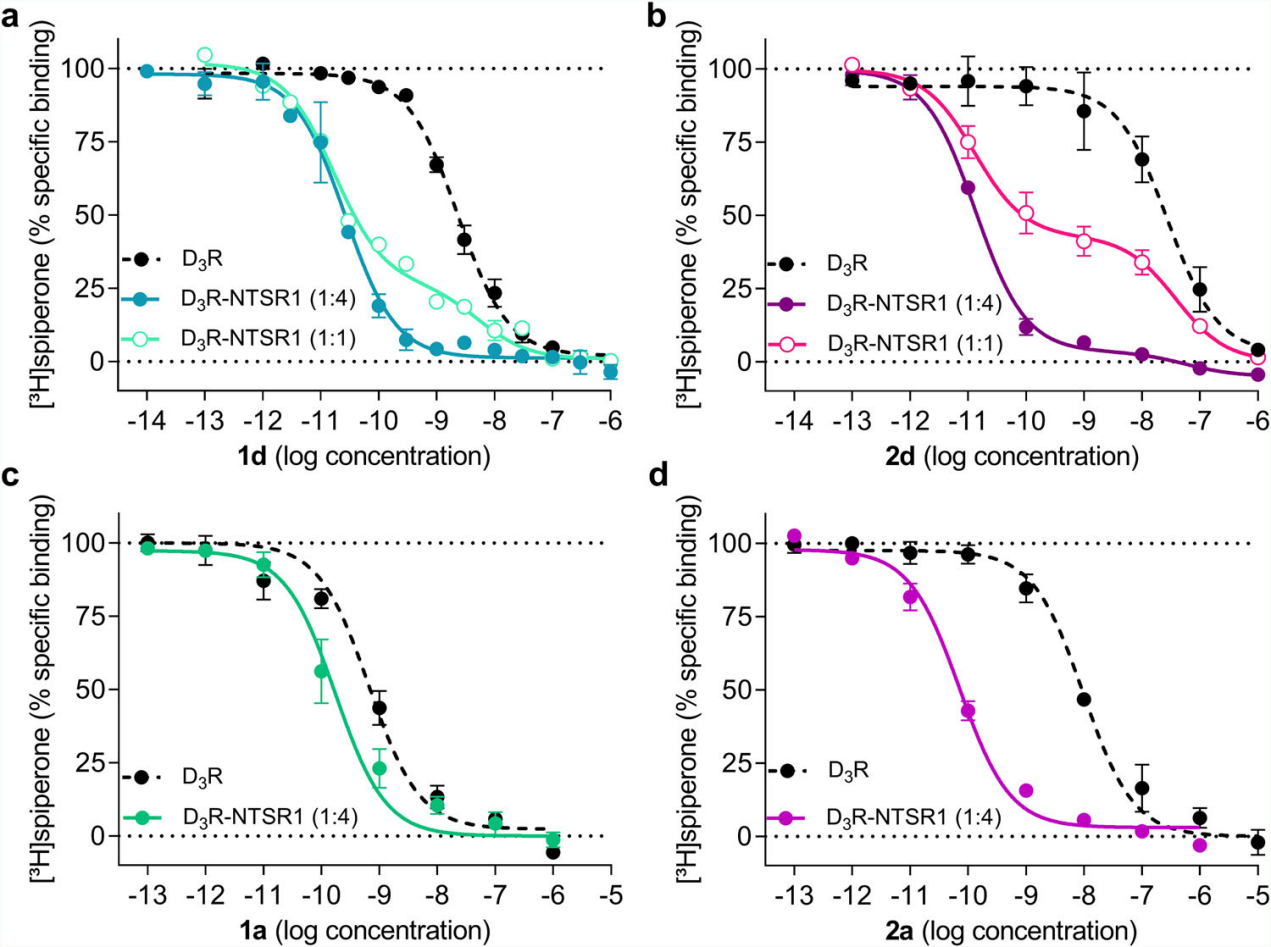
D_3_R Binding behaviour of bivalent ligands. Competition binding experiments were carried out with [³H]spiperone and membranes from HEK293T cells either monoexpressing D_3_R or coexpressing D_3_R and NTSR1. Bivalent ligands **1d** (**a**) and **2d** (**b**) comprising the long 88-atom spacer or **1a** (**c**) and **2a** (**d**) comprising the short 22-atom spacer bind to D_3_R with binding affinities in the high picomolar to low nanomolar range (*n* = 4 for **1d** and *n* = 3 for **1a**, **2a**, **2d**). Coexpression of an excess of NTSR1 (relative stoichiometry D_3_R to NTSR1 1:4) results in a 5.5- to 1460-fold increase in affinity (*n* = 4 for **1a**; *n* = 5 for **1d**, **2d** and *n* = 3 for **2a**). Biphasic displacement curves revealing two distinct affinities corresponding to the high- and low-affinity binding mode are resolved when experiments are carried out at a 1:1 D_3_R to NTSR1 stoichiometry (*n* = 3 for **1d** and **2d**). Data are presented as mean ± s.e.m. of *n* biologically independent experiments.

We have recently developed bivalent ligands which are able to recognise D_2_R-NTSR1 heterodimers with subnanomolar affinity and high selectivity over monomeric D_2_R and moderate selectivity over monomeric NTSR1^11^. Chemically, these ligands are composed of a dopamine receptor agonist (*N*-propylindanyl-2-amine) or antagonist (2-methoxy-phenylpiperazine) that is connected to the peptide NTSR1 agonist NT(8-13) by a flexible linker of variable size resulting in the probe molecules **1a-d** and **2a-d**, respectively. This linker consists of a hydrophilic PEG-derived spacer of variable size (22–88 atoms, *m* = 1–4, Fig. 1b) that is connected to the agonist or antagonist pharmacophore through a biphenyltriazole moiety. To investigate whether these bivalent ligands can target D_3_R-NTSR1 complexes, we performed radioligand binding experiments with membranes from D_3_R mono- and D_3_R-NTSR1 coexpressing HEK293T cells (Supplementary Table 1). In membranes from HEK293T cells expressing only the D_3_R, all ligands were able to displace [^3^H]spiperone with *K*_i_ values ranging from 0.28 nM to 0.85 nM for compounds **1a**-**d** and 4.4 nM to 9.5 nM for compounds **2a**,**c**,**d**. These affinities are in a similar range to those previously reported for binding to monoexpressed NTSR1^11^ (0.24–2.6 nM, Supplementary Table 1). Upon coexpression of D_3_R with an excess of NTSR1, the affinity of all compounds strongly increased (relative receptor stoichiometry D_3_R/NTSR1 1:4, Supplementary Table 1). While the spacer length had no effect on D_3_R binding affinity in monoexpressing membranes, the affinity for coexpressing membranes slightly improved with an increasing spacer length. The highest affinities were observed for ligands **1d** and **2d**, displaying *K*_*i*_-values in the one-digit picomolar range (6.1 ± 3.2 pM for **1d**; 4.8 ± 0.8 pM for **2d**, Fig. 2a, b). Due to their lower affinities for monoexpressed D_3_R, bivalent ligands bearing a dopaminergic antagonist possess higher selectivity for the D_3_R-NTSR1 coexpressing membranes (**2a**, **c**, **d**; 220–1460-fold compared to D_3_R, 16–170-fold compared to NTSR1) than type **1** bivalent dopamine receptor agonists (**1a**-**d**; 5.5–140-fold compared to D_3_R, 22–93 fold compared to NTSR1, Supplementary Table 1). Importantly, coincubation with an excess of NT(8-13) (1 µM) and consequently prevention of a bivalent binding mode by means of a NTSR1-blockade (Supplementary Fig. 2a–c) strongly affected high-affinity binding, leading to *K*_*i*_-values that were comparable to those of D_3_R monoexpressing cells (Supplementary Table 1).

It should be noted, that radioligand displacement curves were predominantly monophasic, with more than 90% of binding to the D_3_R occurring at very low ligand concentration, if NTSR1 was coexpressed in excess relative to D_3_R (4:1 receptor stoichiometry). In contrast, biphasic curves revealing two distinct binding affinities were resolved when membranes from cells expressing an equal amount of NTSR1 and D_3_R were used (1:1 receptor stoichiometry, Fig. 2a, b). Under these conditions, 73 ± 1% (**1d**) or 58 ± 1% (**2d**) of radioligand displacement occurred in low picomolar ligand concentration, respectively. This high-affinity binding component was depleted in presence of 1 µM NT(8-13) (Supplementary Fig. 2d), indicating that high-affinity binding is the result of a bivalent D_3_R-NTSR1 binding mode and low-affinity binding is the result of monovalent engagement of the D_3_R or NTSR1, respectively.

Interestingly, an increase in D_3_R-binding affinity upon coexpression of NTSR1 was also observed for compounds **1a** and **2a** comprising a short 22-atom linker (Fig. 2c, d). Similar to ligands with longer spacers, this high-affinity binding was blocked by 1 µM NT(8-13) (Supplementary Fig. 2e). These results are surprising because ligands with such short spacers did not show higher affinity binding to D_2_R-NTSR1 coexpressing membranes, expected for bivalent binding^11^. This indicates that in contrast to the D_2_R-NTSR1 dimer, one spacer unit (22 atoms, *m* = 1, Fig. 1b) is sufficient to allow binding to both D_3_R and NTSR1 protomers of D_3_R-NTSR1 heterodimers.

### BRET saturation experiments reveal influences of bivalent ligands on D_3_R-NTSR1 interaction

**Fig. 3:**
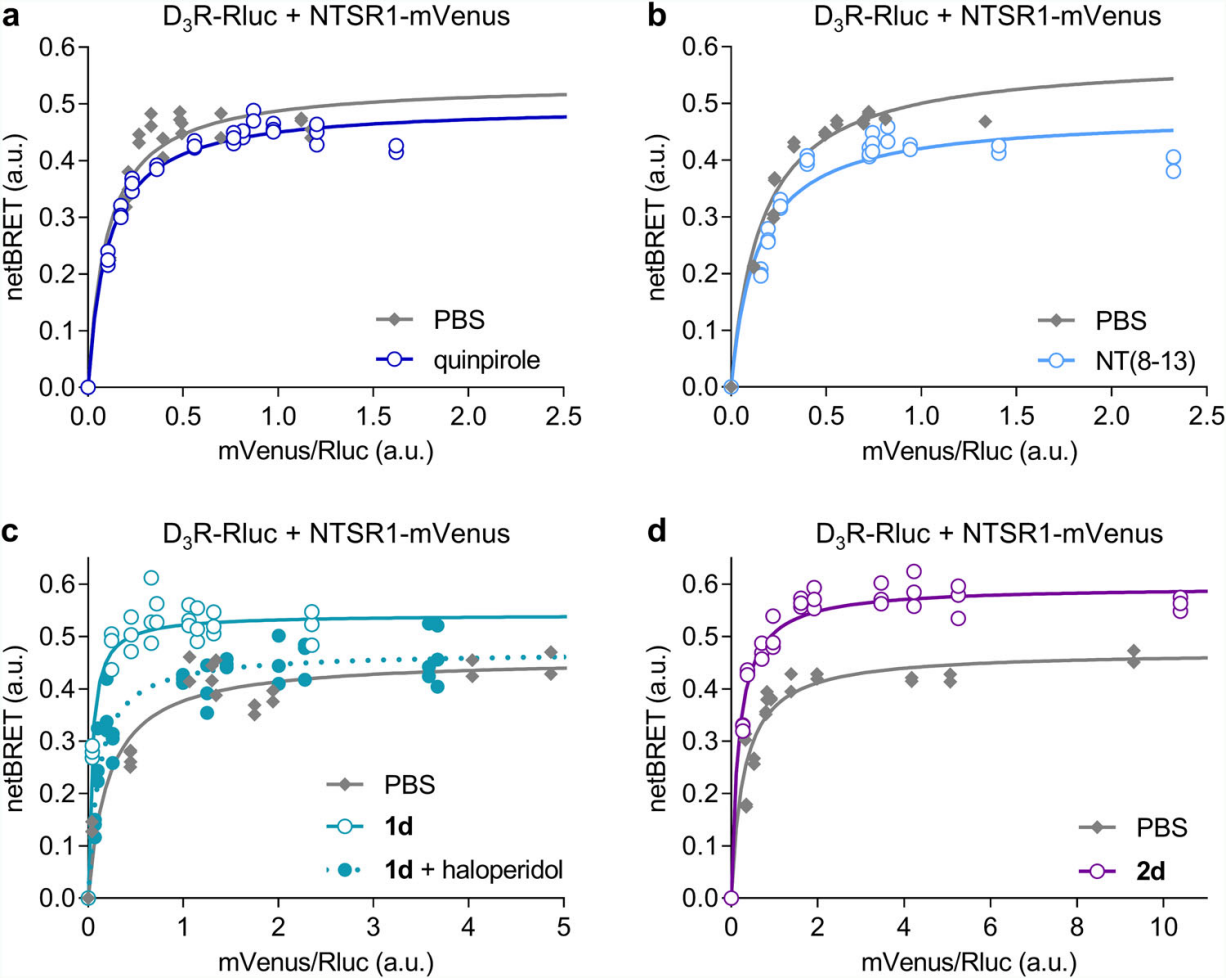
BRET Saturation curves are influenced by mono- and bivalent ligands. **a**, **b** The hyperbolic BRET saturation curve for the coexpression of D_3_R-Rluc and NTSR1-mVenus is indicative of a specific protein–protein interaction. Stimulation of either receptor with monovalent agonists (1 µM) reduces BRET_max_. **c**, **d** The presence of bivalent ligands (10 nM) strongly increases BRET_max_ and slightly reduces BRET_50_. Preincubation with haloperidol (10 µM, 30 min) abolishes the effect. Data show individual replicates for a single representative experiment of at least three independent repetitions.

**Fig. 4:**
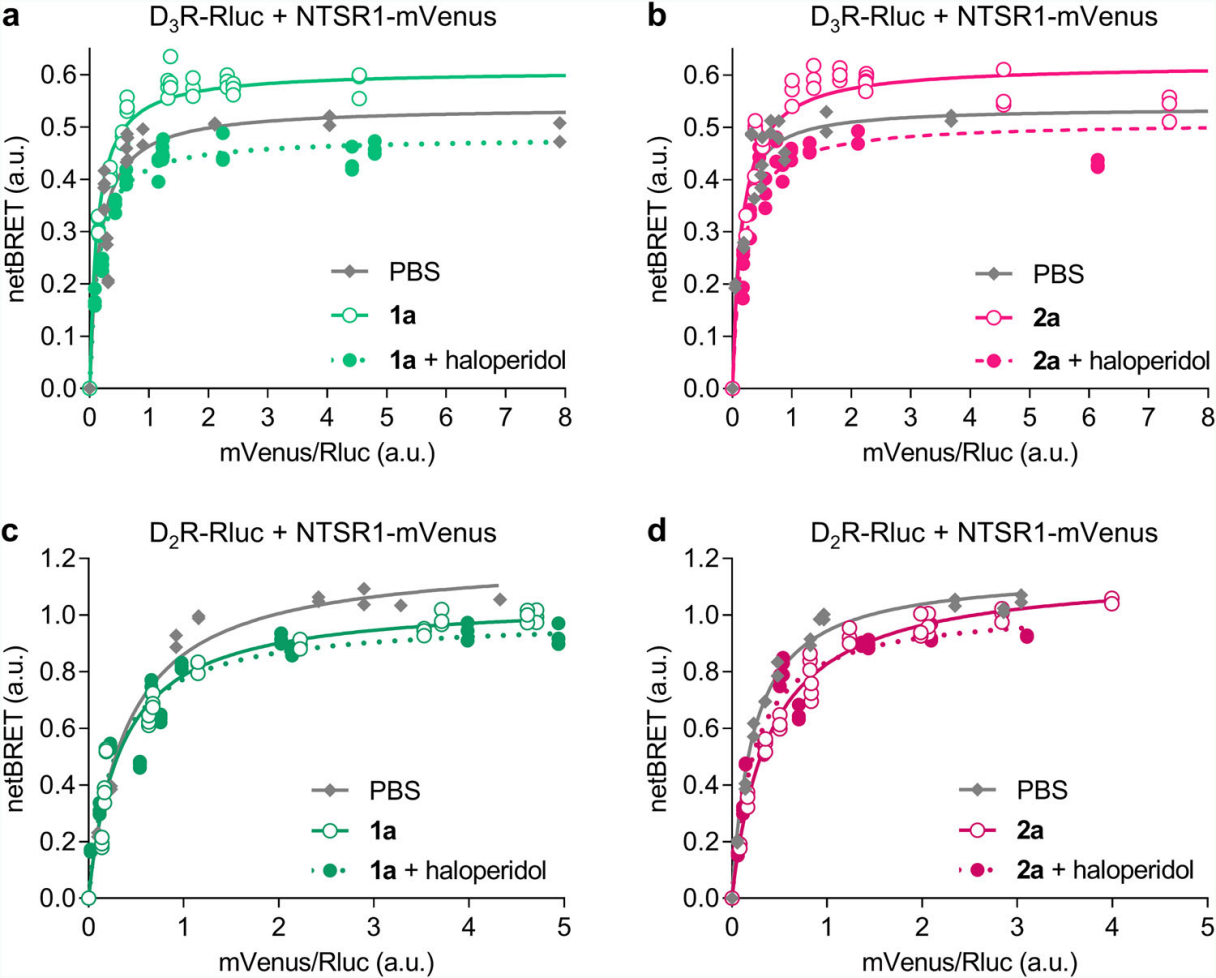
Short-spacer bivalent ligands selectively act on D_3_R-NTSR1. **a**, **b** Bivalent ligands with a 22-atom spacer (**1a**, **2a**) increase BRET_max_ between D_3_R-Rluc and NTSR1-mVenus, but not D_2_R-Rluc and NTSR1-mVenus (**c**, **d**). Preincubation with haloperidol (10 µM, 30 min) prevents the effect of the bivalent ligand, resulting in a decrease in BRET_max_ comparable to incubation with NT(8-13) alone. Data show individual replicates for a single representative experiment of at least three independent repetitions.

In order to directly investigate the effect of ligand binding on the formation of D_3_R-NTSR1 heterodimers, we performed BRET titration experiments using D_3_R fused to *Renilla* luciferase (Rluc) and NTSR1 tagged with the mVenus fluorescent protein, in analogy to previous studies investigating the interaction between NTSR1 and D_2_R.^30,34,37,43^ With increasing levels of NTSR1-mVenus expression, a hyperbolic saturation curve was obtained, pointing towards a specific interaction of D_3_R and NTSR1 (Fig. 3a). Proximity of the two receptors was also observed using in situ proximity ligation assays (PLA)^44^ in HEK293T cells transiently transfected with wild-type D_3_R and NTSR1 (Supplementary Methods and Supplementary Fig. 3). While preserving the hyperbolic shape of the BRET saturation curve, 1 µM of the monovalent dopamine and neurotensin receptor agonists quinpirole and NT(8-13), respectively, led to a decrease in the maximum BRET signal (BRET_max_, Fig. 3a, b) for the D_3_R-Rluc/NTSR1-mVenus complex. In contrast, all bivalent ligands (10 nM) substantially increased BRET_max_ and lowered BRET_50_ and thus promoted the interaction of the two receptors (Figs. 3c, d, 4a, b, Supplementary Fig. 4 and Supplementary Table 2). Similar results were observed when Rluc was exchanged for the brighter and smaller nanoluciferase^45^ (Nluc, Supplementary Fig. 5a). The increase in BRET_max_ was most pronounced upon incubation with the bivalent ligands **1d** and **2d** comprising the long 88-atom spacer indicating a linker-dependent effect. Blockade of the D_3_R by preincubation with high concentrations of the antagonist haloperidol (10 µM, 30 min), and thus inhibition of the bivalent binding mode, completely abolished the effect of the bivalent ligands on BRET_max_ (Figs. 3c and 4a, b). In agreement with previous investigations of D_2_R-NTSR1 heterodimers^11^, bivalent ligands **1a** and **2a** with the short 22-atom spacer did not lead to an enhanced protein-protein interaction in D_2_R-NTSR1 coexpressing cells. Instead, a decrease in BRET_max_ similar to the effect of monovalent NT(8-13) was observed (Fig. 4c, d). In contrast, incubation with **1a** or **2a** led to an increase in BRET_max_ and a decrease in BRET_50_ in cells expressing NTSR1 together with the D_3_R subtype (Fig. 4a, b), confirming the selectivity of the short-spacer ligands for the D_3_R-NTSR1 dimer. Consequently, preincubation with haloperidol had no influence in the D_2_R-NTSR1 system, while it reverted the effects of the bivalent ligands in the D_3_R-NTSR1 system (Fig. 4a–d).

BRET displacement experiments^46^ with wild-type NTSR1 further confirmed that the effects on BRET_max_ observed in BRET saturation experiments with D_3_R-Rluc and NTSR1-mVenus are due to changes in the interaction of the two receptors. When an increasing amount of untagged NTSR1 was cotransfected to a constant combination of D_3_R-Rluc and NTSR1-mVenus, the increase or decrease in BRET_max_ (ΔBRET) induced by 10 nM **1d** or 1 µM NT(8-13), respectively, was diminished, indicating that wild-type NTSR1 competes with NTSR1-mVenus for interaction with D_3_R-Rluc (Supplementary Fig. 6a). Cotransfection of increasing amounts of CXCR4, a GPCR devoid of specific interactions with D_3_R^47^, does not reduce the effect of the bivalent ligand **1d** (Supplementary Fig. 6b) and has only minor effects on the change in BRET induced by NT(8-13).

Interestingly, we found no differences between type **1** and type **2** ligands, ruling out possible influences of D_3_R activation on the BRET saturation assays, since ligands **1a**-**d** harbour a dopamine agonist pharmacophore whereas type **2** ligands’ dopamine pharmacophore is an antagonist. Given that neither chemical inhibition of NTSR1 signalling with the the G_q/11_ inhibitor YM254890^48^ (Supplementary Fig. 5c, d), nor knockout of β-arrestins (Δβ-arrestin HEK cells^49^, Supplementary Fig. 5b) affected the ligand-mediated changes in BRET_max_ between D_3_R-Rluc and NTSR1-mVenus, these are unlikely to result from interference of intracellular signalling proteins. Yet, a potential influence of signal transducers on BRET donors and acceptors cannot be fully excluded, since NTSR1 is known for its promiscuous coupling to various types of G proteins^50^.

**Fig. 5:**
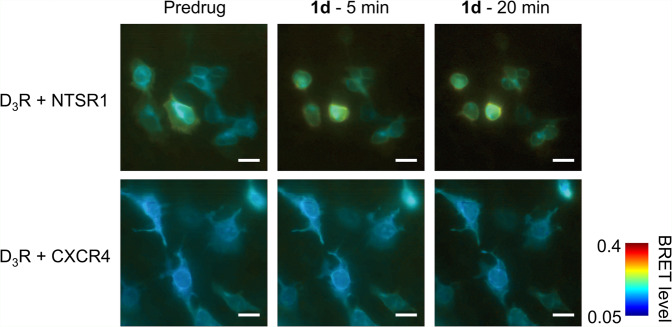
BRET imaging of D_3_R interaction with NTSR1 compared to CXCR4. HEK293SL cells were transfected with BRET donor D_3_R-Nluc and BRET acceptor NTSR1-mVenus or CXCR4-mVenus. 10 µM coelenterazine 400a was added as a substrate. BRET images were obtained before and after the treatment with 10 nM ligand **1d** either for 5 min or 20 min. In each image, BRET levels from 0.05 to 0.4 are expressed as a heat map colour code from blue to red. Adjustments for the correction of the photon counting saturation and Poissonian denoising were applied to the entire images as described in the Methods section. Scale bars, 20 µm. .

To investigate the interaction of D_3_R and NTSR1 at the single cell level, we performed live-cell BRET imaging^51^. Thus, HEK293SL cells were transfected with D_3_R-Nluc as the BRET donor and NTSR1-mVenus as the BRET acceptor. After addition of the BRET substrate, an intense BRET signal was observed in cells coexpressing the two receptors. In contrast, coexpression of D_3_R-Nluc with CXCR4-mVenus resulted in a very weak BRET signal (Fig. 5). Treatment with the bivalent ligand **1d** (10 nM) further increased BRET only for the D_3_R-NTSR1 coexpressing cells (Fig. 5 and Supplementary Movie 1), which is consistent with the bivalent ligand acting on the D_3_R-NTSR1 dimer, either by promoting/stabilising the dimer itself or changing the relative receptor conformations. Although it cannot be completely excluded that changes in cell shape occurring upon ligand addition influence the resulting BRET signal, it is unlikely an imaging artefact since BRET is a ratiometric measurement.

### Engaging D_3_R-NTSR1 heterodimers results in unique D_3_R trafficking

**Fig. 6:**
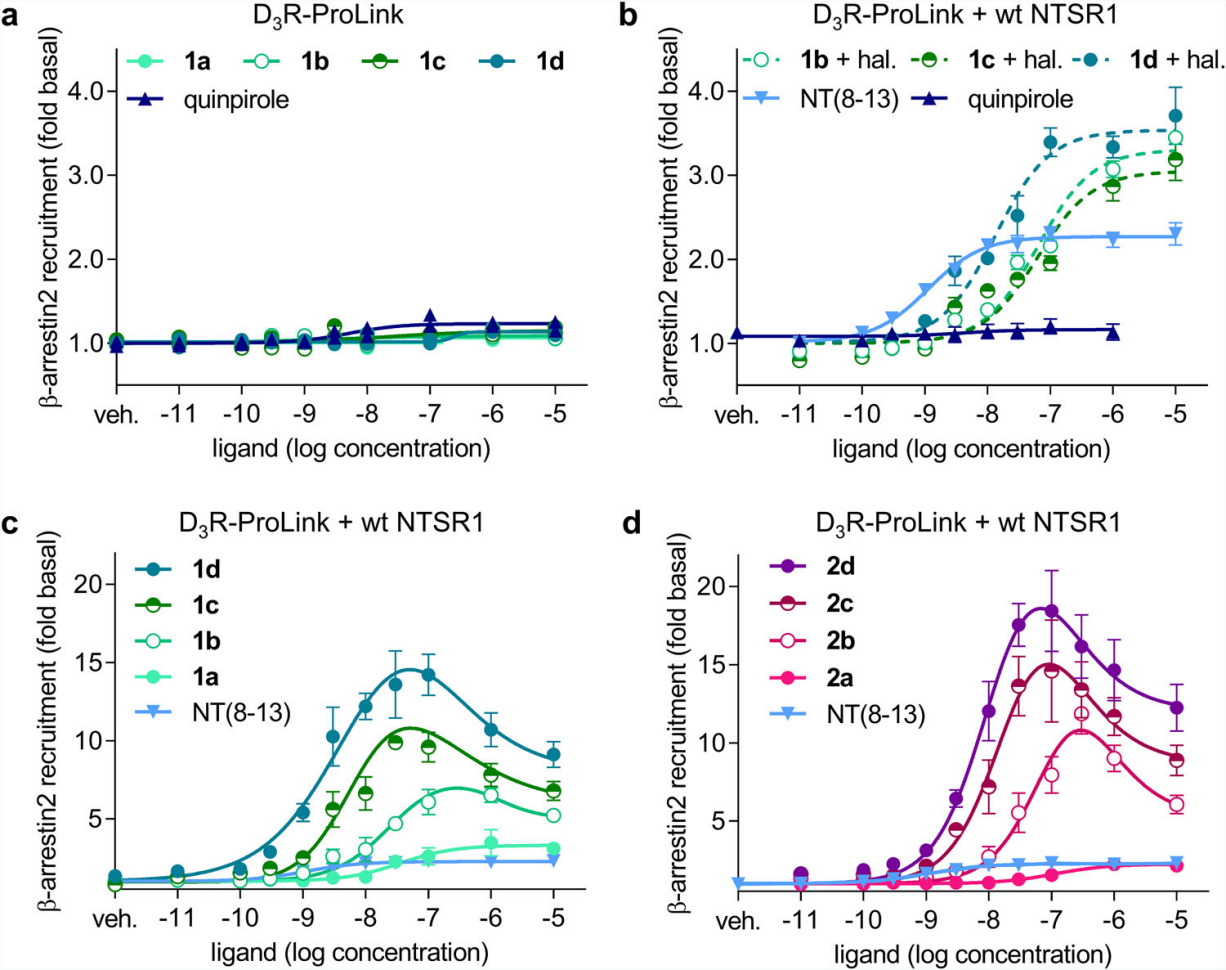
β-arrestin2 recruitment to D_3_R mono- and D_3_R-NTSR1 coexpressing HEK293 cells indicates a specific effect of the bivalent ligands. The DiscoverX enzyme fragment complementation assay allows detection of β-arrestin2 recruitment to a GPCR that is C-terminally tagged with the ProLink fragment. **a** No β-arrestin2 recruitment to D_3_R-ProLink was detected after stimulation with quinpirole or compounds **1a**-**d** (individual data points, *n* = 2 independent experiments). **b** Coexpression of wild-type NTSR1 does not alter β-arrestin2 recruitment to D_3_R-Prolink upon stimulation with quinpirole (*n* = 3). NT(8-13) induces a concentration dependent response (EC_50_ 1.5 ± 0.4 nM, *n* = 8). **c**, **d** Stimulation with type **1** and **2** bivalent ligands results in bell-shaped concentration-response curves in D_3_R-ProLink-NTSR1 coexpressing cells (*n* = 4 for **1a**-**c**, **2b** and *n* = 3 for **1d**, **2a**,**c**,**d**). **b** Prevention of the bivalent binding mode by high concentrations of haloperidol (1 µM, 30 min preincubation, *n* = 3) reduces β-arrestin2 recruitment close to the level of NT(8-13) alone. Error bars denote s.e.m. of *n* independent experiments.

Even though the D_3_R possesses high sequence homology with the D_2_R^12^, it is known for its weak interactions with β-arrestins, as well as negligible agonist-induced endocytosis^52,53^. In contrast, functional studies^50^ and the recently published cryo-EM structures of the NTSR1-β-arrestin complexes^54,55^ leave no doubt about the ability of NTSR1 to recruit β-arrestin and to form a high-affinity complex. To determine the effect of heterodimerization between the D_3_R and NTSR1, we investigated β-arrestin recruitment in HEK293 cells stably expressing β-arrestin2 fused to an engineered galactosidase using enzyme fragment complementation (DiscoverX Pathhunter). When the complementary ProLink tag was fused to the C-terminus of D_3_R (D_3_R-ProLink), neither bivalent ligands **1a**-**d** comprising the agonistic D_3_R pharmacophore, nor the reference agonist quinpirole were able to induce detectable β-arrestin2 recruitment in cells expressing only D_3_R-ProLink (Fig. 6a), although these ligands displayed agonist properties for D_3_R-mediated G protein activation (Supplementary Fig. 7). When wild-type NTSR1 was coexpressed together with D_3_R-ProLink, stimulation with monovalent NT(8-13) resulted in a concentration dependent (EC_50_ 1.5 ± 0.4 nM) recruitment of β-arrestin2 indicated by a 2.3-fold increase of the basal luminescence signal (Fig. 6b). It should be noted that in this setup, recruitment of β-arrestin2 to NTSR1 is only detected if it occurs in sufficient proximity to allow for enzyme complementation^11^ with the D_3_R carrying the ProLink enzyme fragment. When D_3_R-NTSR1 coexpressing cells were incubated with the bivalent ligands, bell-shaped concentration-response curves were observed. The maximum signal elicited by the bivalent ligands greatly exceeded the response to monovalent NT(8-13). In agreement with the results from the BRET saturation experiments probing dimerization, the effect was most pronounced for compounds with the long 88-atom spacer, **1d** (E_max_ 14-fold of basal luminescence) and **2d** (E_max_ 18-fold of basal luminescence, Fig. 6c,d). Comparison of type **1** and **2** bivalent ligands revealed that the presence of an agonistic D_3_R pharmacophore is not required for the induction of β-arrestin2 recruitment, as type **1** bivalent ligands do not show superior efficacy compared to type **2** bivalent ligands comprising the D_3_R antagonist. Hence, β-arrestin recruitment is achieved by activation of NTSR1 through the NT(8-13) fragment of the bivalent ligands. Importantly, inhibition of the bivalent binding mode by addition of the D_3_R antagonist haloperidol (1 µM) almost completely prevented the increase in the maximum effect and abolished the bell-shape, leading to regular concentration-response curves for the bivalent ligands (Fig. 6b). When β-arrestin2 recruitment was investigated with the NTSR1 fused to the ProLink tag (NTSR1-ProLink), the monovalent agonist NT(8-13) was found to strongly induce β-arrestin2 recruitment with a potency in the subnanomolar range (EC_50_ 0.58 ± 0.06 nM, mean ± s.e.m., *n* = 8). Stimulation of NTSR1-ProLink with the bivalent ligands **1b**-**d** and **2b**-**d** also led to sigmoid concentration-response curves with full agonist efficacy, but up to 25-fold lower potency compared to the monovalent agonist NT(8-13) (Supplementary Fig. 8a, b). Similar results were also found using BRET-based arrestin recruitment assays^56^ (Supplementary Methods and Supplementary Fig. 8c-f). This is in good agreement with NTSR1-mediated G_q_ protein activation, where all bivalent ligands showed full agonist efficacy, but were 6–16-fold less potent than NT(8-13) (Supplementary Table 3 and Supplementary Fig. 9a, b). Coexpression of D_3_R marginally improved the potency of the bivalent ligands on the G_q_ response (Supplementary Table 3 and Supplementary Fig. 9c, d).

**Fig. 7:**
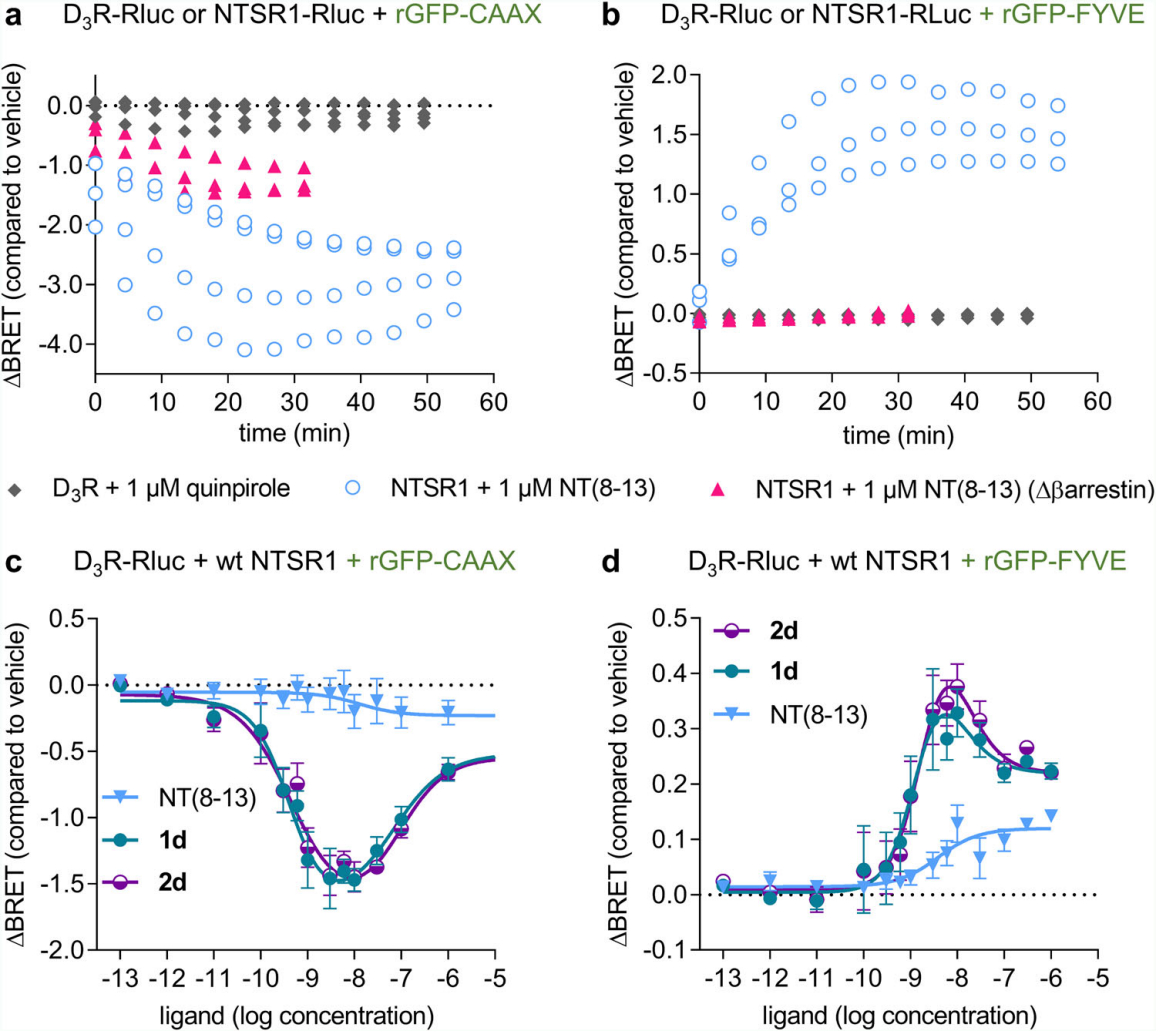
Bystander BRET shows the bivalent ligands’ ability to induce cointernalization of D_3_R-NTSR1. Bystander BRET between D_3_R-Rluc or NTSR1-Rluc and rGFP-CAAX as membrane marker or rGFP-FYVE as endosome marker is used to monitor receptor trafficking. **a**, **b** Time course of receptor internalisation. Stimulation of NTSR1 with NT(8-13) results in time-dependent sequestration and internalisation as detected by a decreased BRET signal between Rluc and rGFP-CAAX (*n* = 4) and an increased BRET signal between Rluc and rGFP-FYVE (*n* = 3). In cells devoid of β-arrestin1 and β-arrestin2, **a** a small fraction of NTSR1 (*n* = 3) is sequestered from the cell membrane, **b** but does not translocate into FYVE-labelled endosomes (*n* = 3). Internalisation of the D_3_R is not observed (*n* = 4). Data are displayed as individual results from biologically independent experiments. **c**, **d** Stimulating D_3_R-Rluc-NTSR1 coexpressing cells with the bivalent ligands results in D_3_R trafficking from the membrane to endosomes. NT(8-13) is able to induce a small increase in the endosomal compartment. Concentration-response curves for D_3_R internalisation reveal a bell-shaped profile for bivalent ligands **1d** and **2d** but not for monovalent NT(8-13). Data show mean ± s.e.m. of **c** *n* = 6 and **d** *n* = 5 independent experiments. .

Application of rGFP targeted to the plasma membrane using a prenylation sequence (CAAX) or to the endosome using a FYVE domain enables quantification of receptor sequestration and internalisation of a GPCR tagged with Rluc by enhanced bystander BRET^56^. In accordance with previous findings^52^, we could not detect internalisation of monoexpressed D_3_R-Rluc after stimulation with 1 µM quinpirole (Fig. 7a, b) or the bivalent ligands **1d** or **2d**, representative for the D_3_R agonist or antagonist ligand series (10 nM, Supplementary Fig. 10a, c). In contrast, as indicated by a decreasing BRET signal between the receptor and rGFP-CAAX and an increasing signal between the receptor and rGFP-FYVE, a strong time-dependent endocytosis of monoexpressed NTSR1-Rluc was observed upon stimulation with NT(8-13) (Fig. 7a, b). Similar to NT(8-13), representative bivalent ligands **1d** and **2d** caused a concentration dependent internalisation of NTSR1 (Supplementary Fig. 10b, d). This is not surprising, since all three ligands share the NT(8-13) substructure. In agreement with earlier findings demonstrating the importance of β-arrestins for internalisation and intracellular trafficking^57^, this increase of BRET between rGFP-FYVE and NTSR1-Rluc was completely abolished and the decrease of BRET between rGFP-CAAX and NTSR1-Rluc was strongly diminished, but not absent, in β-arrestin knockout cells (Fig. 7a, b). It is possible that the polybasic sequence of the CAAX-domain does not allow a completely homogenous distribution throughout all compartments of the plasma membrane. Charged residues could impair its diffusion into more ordered hydrophobic regions such as lipid rafts. Stimulation of NTSR1 results in G protein activation, conformational changes and receptor modifications that may lead to receptor sequestration and redistribution to hydrophobic compartments^58^ possibly resulting in reduced bystander BRET between NTSR1-Rluc and rGFP-CAAX. Interestingly, bivalent ligands were able to induce D_3_R endocytosis, when wild-type NTSR1 was coexpressed with the tagged D_3_R-Rluc (Fig. 7c, d and Supplementary Fig. 11a, b). Under these conditions, the monovalent agonist quinpirole still did not elicit an effect (Supplementary Fig. 11a, b), while NT(8-13) slightly increased BRET between the receptor and rGFP-FYVE (Fig. 7d and Supplementary Fig. 11b), but did not affect surface BRET between D_3_R-Rluc and rGFP-CAAX (Fig. 7c and Supplementary Fig. 11a). Concentration-response curves for the bivalent ligands **1d** and **2d** again revealed a bell-shaped concentration-response relationship, with strongly enhanced maximum efficacy compared to NT(8-13) (Fig. 7c, d). Similar to the results from the β-arrestin2 recruitment assays, D_3_R cointernalization depended on the spacer length, but not the nature of the D_3_R pharmacophore (Supplementary Fig. 11a, b). As for monoexpressed NTSR1, the knockout of β-arrestins strongly diminished the effect of the bivalent ligands on D_3_R endocytosis as assessed by coexpressing D_3_R-Rluc, wild-type NTSR1 and rGFP-CAAX or rGFP-FYVE, emphasising that the internalisation of D_3_R is in fact driven by activation and internalisation of NTSR1 (Supplementary Fig. 11c, d). Together these findings are indicative for a specific effect of the bivalent ligands at low ligand concentration. Under these conditions, the ligands simultaneously engage the two protomers of the D_3_R-NTSR1 complex, enhance the protein-protein interaction and activate NTSR1, thereby leading to internalisation of the trimeric ligand-receptor complex. At higher ligand concentration, monovalent binding prevails and the effect of the bivalent ligands resembles monomeric NT(8-13), hence explaining the bell-shape of the concentration-response curves.

Results from BRET-based endocytosis assays were confirmed by surface ELISA (Supplementary Methods). As expected, surface expression of monoexpressed NTSR1 was reduced to 56 ± 4% (mean ± s.e.m., *n* = 6) with 1 µM of NT(8-13) and 72 ± 5% (mean ± s.e.m., *n* = 5) with 10 nM of compound **1d**, but not substantially influenced by 1 µM quinpirole (87 ± 6%, mean ± s.e.m., *n* = 3, Supplementary Fig. 12a). Again, treatment with 1 µM of quinpirole, 1 µM of NT(8-13) or 10 nM of bivalent compound **1d** did not lead to apparent changes in surface expression of monoexpressed D_3_R (Supplementary Fig. 12b). Upon coexpression of NTSR1, stimulation with NT(8-13) reduced surface expression of the D_3_R to 85 ± 6% (mean ± s.e.m., *n* = 3) of the control conditions. Stimulation with the bivalent ligand **1d** led to even stronger internalisation of D_3_R in the D_3_R-NTSR1 coexpressing cells (67 ± 5%, of surface receptor remaining, mean ± s.e.m., *n* = 3, Supplementary Fig. 12c). In agreement with the BRET experiments, the influences on surface expression were abolished in Δβ-arrestin HEK cells (Supplementary Fig. 12d).

## Discussion

Specific interactions of class A GPCRs within the plasma membrane of living cells are capable of modulating the signalling properties, binding affinities and trafficking of the individual receptor protomers^1–6^. While the dimerization of class C GPCRs such as the GABA_B_ or metabotropic glutamate receptors is compulsory for their expression and function^59^, the formation of multimeric class A GPCR complexes is known to be transient^60–63^. Diffusion of class A GPCRs within the membrane can result in random collision or specific interactions of the protomers. Hence, they exist in an equilibrium between monomeric and oligomeric states, which interchange frequently and can be affected through ligand binding^61,64,65^. Bivalent ligands that are composed of two pharmacophores connected by a linker of suitable size are able to engage the binding pockets of two receptors simultaneously. Consequently, bivalent ligands can either recognise a preformed receptor complex, such as a homo- or heterodimer^66^, or sequentially bind one protomer and increase the local concentration of the second receptor by binding of the second tethered pharmacophore^7^. Irrespective of the question whether class A GCPR dimerization has functional consequences per se, the mere coexpression of two receptors can be exploited by heterobivalent ligands to harness the thermodynamic advantage of the bivalent binding mode over monovalent binding to either receptor, conferring high affinity and selectivity for the receptor dimer^7,11^

D_3_R and NTSR1, two receptors that are involved in various neuropsychiatric diseases and connected to addiction, show overlapping expression in the CNS, especially in the islands of Calleja, a brain region that is connected to reward seeking behaviour^18^. Moreover, neurotensin has been shown to influence D_3_R agonist binding in a G protein independent manner in rat brain tissue^38^, and D_3_R and neurotensin mRNA expression overlap in the rat *nucleus accumbens*, although direct evidence for a cellular colocalization and direct interaction of D_3_R and NTSR1 in vivo is pending.

Employing a set of previously described bivalent ligands^11^ in binding studies with the radioligand [^3^H]spiperone, we observed that the coexpression of NTSR1 and D_3_R leads to a substantial increase in binding affinity compared to cells that only express D_3_R (up to 1460-fold) or NTSR1 (up to 170-fold). This effect depends on the spacer length, leading to one-digit picomolar affinities for ligands **1d** and **2d** with an 88-atom PEG-spacer. The high-affinity binding of the ligands in the D_3_R-NTSR1 coexpressing cells can be attributed to a bivalent binding mode, as it is abolished in presence of high concentration (1 µM) of monovalent NT(8-13), and the proportion of high- and low-affinity binding sites is affected by the stoichiometry of receptor expression. Compared to the previously reported binding profiles of bivalent ligands to D_2_R-NTSR1^11^, the proportion of high-affinity binding sites reflecting the heterodimer-ligand complex appears to be higher in D_3_R-NTSR1 coexpressing cells.

BRET-based methods are powerful in detecting and characterising protein-protein interactions^67^. Here we employed BRET saturation experiments and live-cell BRET imaging to study the influence of monovalent and bivalent ligands on the interactions of D_3_R and NTSR1. In agreement with previous findings from single molecule tracking experiments describing a prolonged lifetime of D_2_R homodimers upon bivalent ligand binding^61^, our results indicate that bivalent ligands stabilise the D_3_R-NTSR1 interaction. This leads to an increased proportion of dimers, regardless of whether a preformed heterodimer is addressed, or if heterodimerization is a bivalent ligand-induced effect. The observation that the increase in BRET_max_ can be abolished by blocking the D_3_R with haloperidol and by titration of wild-type NTSR1 ensures that the observed effects are the result of specific bivalent binding to both receptors. Marsango *et al*. previously reported reduced levels of D_3_R homodimers after incubation with spiperone and haloperidol^68^, while an increase in the proportion of D_2_R and D_3_R homodimers or their lifetime was reported upon incubation with agonists^61,65^. Similar effects seem possible for the D_3_R-NTSR1 receptor pair, where we observed that binding of monovalent agonists disturbs the heteromeric receptor-receptor interaction.

The bivalent compounds of type **1** and **2** comprise dopaminergic pharmacophores that are not subtype-selective between D_2_R and D_3_R. Based on results from BRET saturation and radioligand binding, bivalent ligands **1a** and **2a** with the short 22-atom spacer (m = 1) stabilise D_3_R-NTSR1 heterodimers, while it was previously described that at least 44-atom-spacers (*m* = 2) are required for bivalent binding to D_2_R-NTSR1 heterodimers^11^. The individual quaternary structures of the heterodimers allow bivalent ligands to distinguish between D_3_R-NTSR1 and D_2_R-NTSR1 heterodimers and may leverage an appealing strategy to design subtype selective ligands without the need for using subtype-selective pharmacophores. Because the dimer-selective binding of **1a** and **2a** over D_3_R and NTSR1 monomers is less pronounced compared to their analogs with longer linkers, further optimisation of the linker unit may be of interest for future lead optimisation. Moreover, the combination of D_3_R-selective pharmacophores with longer linkers may be an interesting alternative for the development of heterobivalent ligands for D_3_R-NTSR1 with subtype selectivity over D_2_R-NTSR1.

Previous reports described very weak interactions of the D_3_R with β-arrestins and a PKC-mediated clathrin-dependent lysosomal degradation without the involvement of a GRK2/β-arrestin complex^52,69,70^. In accordance with these findings, we observed that while being able to signal through G proteins, monoexpressed D_3_R does not recruit β-arrestin2 or internalise upon stimulation with quinpirole. The NTSR1 on the other hand, expectedly recruits β-arrestin2 to the plasma membrane and to endosomes and rapidly internalises upon agonist binding. A luminescence signal resulting from stimulation of NTSR1 with NT(8-13) in D_3_R-ProLink-NTSR1 coexpressing cells demonstrates that the proximity of the two receptors is sufficient to enable enzyme complementation. Irrespective of the D_3_R pharmacophore, bivalent ligands lead to β-arrestin recruitment through activation of the NTSR1 within the heterodimeric complex, with much higher efficacy than the monovalent agonist NT(8-13). If bivalent ligands are used in a concentration range where monovalent binding predominates or if bivalent binding is prevented, β-arrestin recruitment and internalisation of only the NTSR1 occurs. This results in a reduced luminescence output and a reduced bystander BRET as indicated by the decline in the terminal phase of the bell-shaped concentration-response curves. Importantly, the stabilisation of the D_3_R-NTSR1 heterodimer with the bivalent ligands enables internalisation of a complex consisting of bivalent ligand, D_3_R, NTSR1 and β-arrestin2, which remains assembled in intracellular compartments. As a result, trafficking of the D_3_R is now driven by NTSR1, dramatically changing its pharmacological properties. In a very recent study, a possible link between receptor dimerization, β-arrestin recruitment and internalisation has also been observed for μ-opioid receptors^63^.

In agreement with our previous findings on the D_2_R-NTSR1 heterodimer^11^, we could not detect any influences on the signalling properties of the NTSR1 upon coexpressing the D_3_R when we investigated β-arrestin2 recruitment and coupling to G_q_ proteins. It should be noted that under the experimental conditions applied, expression levels of NTSR1 are usually higher than those of D_3_R. Hence, the presence of monomeric or homodimeric NTSR1 is to be expected, whose signalling may potentially obscure a dimer-specific signalling effect of the bivalent ligands.

Even though the question whether signalling properties of the D_3_R change upon reaching intracellular compartments remains elusive, we could successfully demonstrate that bivalent ligands are able to specifically address the D_3_R in coexpression with the NTSR1 and that bivalent ligands can shift the trafficking of the D_3_R to a β-arrestin mediated endocytosis.

The finding that a class A GPCR-heterodimer can be addressed and stabilised using bivalent ligands to the point that it remains a complex even after internalisation and within intracellular compartments, helps to understand the molecular consequences of receptor dimerization and bivalent ligand binding. Furthermore, it is encouraging and inspiring for the development of novel pharmacological tools, future drugs and innovative therapeutic approaches, in the case of D_3_R-NTSR1 for example in the field of addiction and substance abuse.

## Methods

### In vitro autoradiography

Animal experiments were approved by the local animal protection authorities (Government of Central Franconia, Germany, No. 55.2 2532-2-618-14) and performed at the FAU Erlangen-Nürnberg in accordance with the relevant institutional guidelines and EU regulations. The radioligands ^18^F-**10** and ^177^Lu-**NT127** were synthesised as described previously^39,40^ and formulated in saline solution. Coronal rat brain sections (12 μm, female Sprague-Dawley rat (Charles River); strain: Crl:CD(SD)/outbred; age: 22–23 weeks) were cut on a cryostat microtome (HM550, Microm, Germany) and thaw-mounted on covered glass slides (Histobond). The brain slices were carefully dried at room temperature and preincubated for 15 min in assay buffer (50 mM Tris-HCl, 5 mM MgCl_2_, 50 μM bacitracin, 0.2% BSA, pH 7.4). Afterwards, the sections were incubated in a 50 mL-pot at room temperature for 60 min in assay buffer containing 100 kBq ∙ mL^−1 18^F-**10** and/or 20 kBq ∙ mL^−1 177^Lu-**NT127** in the presence or absence of BP897 (50 nM) or neurotensin (1 µM). Subsequently, slices were washed three times for 2 min in fresh cold assay buffer and dipped briefly in ice-cold distilled water. The slices were dried under a slight stream of air and exposed to a phosphor imager screen (FUJI Imaging Plate BAS-IP SR) overnight and analysed with a high-resolution radioluminography laser scanner (DÜRR Medical HD-CR 35 Bio, Raytest) to visualise the ^18^F-labelled and the ^177^Lu-labelled binding sites. After 24 h and complete decay of ^18^F, the phosphor imager screen was erased and the slices were again exposed to the imager screen for additional 3 days and again analysed with the high-resolution radioluminography laser scanner to visualise the remaining ^177^Lu-labelled binding sites only. Images were created using the software Quantity One (BioRad).

### Cell culture

HEK293T (ATCC accession number CRL-11268, gift from the Chair of Physiology, FAU Erlangen), HEK293SL^71^, Δβ-arrestin-HEK^49^ (both donated from Stephane Laporte, Mc Gill University, Montreal, Canada) and HEK293 cells stably expressing β-arrestin2 tagged to the enzyme acceptor (β-arrestin HEK293, DiscoverX) were cultivated in DMEM supplemented with 10% foetal bovine serum (HEK293T, β-arrestin HEK293 and Δβ-arrestin-HEK) or 10% newborn calf serum (HEK293SL), 100 µg ∙ mL^-1^ penicillin, 100 µg ∙ mL^-1^ streptomycin, 2 mM l-glutamine (and 150 µg ∙ mL^−1^ hygromycin for β-arrestin HEK293) at 37 °C and 5% CO_2_. All cells were regularly confirmed to be free of mycoplasma contamination employing the PCR Mycoplasma Detection Kit (Applied Biological Materials) or luminescence-based MycoAlert Plus Kit (Lonza).

### Plasmids

Wild-type human D_3_R and NTSR1 cDNAs (pcDNA3.1) were obtained from the cDNA resource center (cdna.org). Plasmids (pcDNA3.1) encoding for receptor Rluc8 fusion proteins used in BRET saturation or internalisation assays (D_3_R-Rluc, NTSR1-Rluc) were constructed in analogy to the previously described D_2S_R-Rluc8 fusion protein^72^ employing overlap PCR on the wild-type cDNAs and ligated using the Gibson assembly cloning kit (New England Biolabs). In each case, a 24 amino acid linker (ATGLRSRAQASNSAVDGTAGPVAT) was inserted between the receptor and the luciferase. NTSR1-mVenus has been described previously^34^, and the respective D_3_R-mVenus fusion protein containing the 5 amino acid linker (GGGAS) was cloned in an analogous manner. For the D_3_R-Nluc construct (pcDNA3.1) used in live-cell BRET imaging, the sequence of the D_3_R including the C-terminal 5 amino acid linker (GGGAS) was cloned in frame between the IL6 export signal and the sequence coding for the nanoluciferase enzyme (secretory Nluc, Promega). Plasmids encoding human β-arrestin2-RlucII^73^, HA-CXCR4-mVenus (pIRESP)^74^, rGFP-CAAX^56^ and rGFP-FYVE^56^ have been described previously. For enzyme fragment complementation-based readout of β-arrestin2 recruitment, D_3_R was C-terminally tagged with the ARMS2-PK2 sequence in analogy to previously described procedures for D_2S_R^11^ and cloned into the pCMV ProLink vector (DiscoverX). For NTSR1-ProLink the cDNA was fused in frame with the PK1 sequence (DiscoverX) in an analogous manner. For ELISA experiments, D_3_R N-terminally containing an HA-cleavable sequence and the Flag-Epitope^75^, and NTSR1 N-terminally labelled with the 3xHA-epitope (cdna.org) were employed.

### Radioligand binding

Affinities of the bivalent ligands were determined by radio-ligand displacement in analogy to previously described protocols^76^. In brief, radioligand binding experiments were carried out using membrane preparations of HEK293T cells transiently monotransfected with D_3_R or cotransfected with D_3_R and NTSR1 in a cDNA ratio to yield the below mentioned expression levels using polyethylenimine or TransIT293 in a 3:1 ratio of transfection reagent to DNA. We determined an expression level of 540–1400 fmol ∙ mg^−1^ protein (*K*_D_ 0.13–0.22 nM) protein for D_3_R in the monoexpression system in saturation binding assays using [^3^H]spiperone (specific acitivity 78.8 Ci ∙ mmol^−1^, Perkin-Elmer, Rodgau, Germany). Membrane preparation with a D_3_R: NTSR1 ratio of 1:1 comprised 518 fmol ∙ mg^−1^ protein (*K*_D_ 0.25 nM) D_3_R and 558 fmol ∙ mg^-1^ protein (*K*_D_ 0.79 nM) NTSR1, determined with the radioligands [^3^H]spiperone and [^3^H]NT(8-13) (specific activity 136 Ci ∙ mmol^−1^, Perkin-Elmer, Rodgau, Germany). Coexpressing membranes with an excess of NTSR1 of at least 4-fold ranged from 200 to 600 fmol ∙ mg^−1^ protein (*K*_D_ 0.04–0.24 nM) for D_3_R and 1500–7300 fmol ∙ mg^-1^ protein (*K*_D_ 0.33–2.3 nM) for NTSR1. Non-specific binding was determined using haloperidol (10 µM for D_3_R) and NT(8-13) (10 µM for NTSR1). Competition binding experiments were carried out using a protein concentration of 4–18 µg protein per well for the coexpression systems and 1–12 µg per well for the monoexpression system in a total volume of 200 µL. The protein concentration of each membrane preparation was determined by the method of Lowry^77^ with bovine serum album (Sigma-Aldrich) as standard. All binding experiments were carried out at 37 °C in binding buffer consisting of 50 mM Tris pH 7.4, 5 nM MgCl_2_, 1 mM EDTA, 100 µg ∙ mL^−1^ bacitracin and 100 µg ∙ mL^−1^ soybean trypsin inhibitor. Data were normalised to non-specific and total binding and analysed employing one- or two-site competition binding algorithms implemented in GraphPad Prism for windows, version 6.0 (GraphPad Software, San Diego California, USA, www.graphpad.com).

### BRET saturation

HEK293T cells were detached using Versene (Invitrogen) and diluted to a concentration of 250,000 cells ∙ mL^−1^ in growth medium. Using polyethylenimine (PEI), 300,000 cells were transiently transfected in suspension with a total amount of 1 µg DNA composed of a BRET donor (100 ng D_2S_R-Rluc or 500 ng D_3_R-Rluc) along with different amounts of BRET acceptor (0–800 ng of NTSR1-mVenus) and complemented to 1 µg with single stranded DNA from salmon testis (ssDNA, Sigma Aldrich). For BRET displacement assays, 300,000 cells per condition were transfected in an analogous manner with 500 ng D_3_R-Rluc, 100 ng NTSR1-mVenus, 0–150 ng of non-labelled NTSR1 or CXCR4 plasmids, complemented to 1 µg with ssDNA. Cells were seeded in a white 96-well plate (Greiner Bio one) at a density of 25,000 cells per well and cultivated at 37 °C, 5% CO_2_. After 48 h, the medium was replaced with prewarmed PBS. After 45-60 min at 37 °C, mVenus fluorescence was determined using a CLARIOstar microplate reader (BMG LabTech) with an excitation filter of 497-15 nm and an emission filter of 535-30 nm. Cells were either preincubated for 30 min with 10 µM haloperidol or ligands were added without preincubation in a final concentration of 10 nM (bivalent ligands) or 1 µM (monovalent ligands). After 10 min incubation at room temperature, Colelenterazine-h (Promega, Mannheim, Germany) was added in a final concentration of 5 µM. The BRET_ratio_ was determined after 20 min at room temperature under light exclusion by simultaneous measurement of Rluc and mVenus emission using a filter set of 475-30 nm (donor) and 535-30 nm (acceptor). To determine netBRET, the BRET_ratio_ obtained in the absence of a BRET acceptor was subtracted. NetBRET was plotted against the ratio of mVenus to Rluc counts. Nonlinear regression was performed using the algorithms for one-site specific binding of GraphPad Prism 6.0. For BRET displacement experiments, ΔBRET was calculated as the difference in BRET_ratio_ between the ligand-treated conditions and vehicle-treated controls.

### BRET imaging

BRET signals were imaged using a BRET microscope composed of an inverted microscope (Eclipse Ti-U, Nikon), an optical filter unit (Lambda 10-2, Sutter Instrument) and an EMCCD camera (HNü512, Nüvü cameras) as described previously^51^. HEK293SL cells were seeded 72 h before the measurement on poly-*D*-lysine-coated 35 mm glass bottom dishes (P35GC-1.5-14-C, MatTek) at a density of 2–6 × 10^5^ cells/dish, and transfected at 48 h before the measurement with 100 ng BRET donor (IL6-D_3_R-GGGAS-Nluc), 400 ng BRET acceptor (NTSR1-mVenus or CXCR4-mVenus) and 500 ng ssDNA using X-tremeGENE 9 transfection reagent (Roche). Just before the imaging experiment, cells were washed with Modified Hank’s balanced salt solution (HBSS) (137.9 mM NaCl, 5.33 mM KCl, 1 mM CaCl_2_, 1 mM MgCl_2_, 0.44 mM KH_2_PO_4_, 0.33 mM Na_2_HPO_4_, 10 mM HEPES pH 7.4). The bivalent ligand **1d** (10 nM) and the luciferase substrate coelenterazine 400a (10 µM, NanoLight technologies) were diluted with HBSS. Photon counting frames were recorded with EM gain 3000 and 10–100 ms exposure according to the signal strength. Frames were integrated continuously for 10 s without filter (total luminescence frames), then for 10 s with a band-pass filter (550/80 nm, acceptor frames). Acceptor and total luminescence Images were generated by repeating 15 integration cycles (total exposure time 150 s/channel) and integrating all frames with the same filter settings using MATLAB 2019b. Images were treated with photometric correction^78^ and iterative poisson image denoising^79^ filters. BRET values were calculated by dividing acceptor counts by total luminescence counts pixelwise, and allocated to ‘jet’ heatmap array. The movie was generated using ImageJ 1.52a. Frame rate is 5 frames ∙ s^−1^ and frame interval is 60 s.

### β-arrestin2 recruitment

β-arrestin2 recruitment was investigated employing the Pathhunter assay as described previously^11^ and in analogy to the manufacturer’s protocol (DiscoverX, Fremont, USA). HEK293 stably expressing β-arrestin2 tagged with the enzyme acceptor (DiscoverX) were transiently transfected using TransIT293 (MoBiTec, Goettingen, Germany). In all, 2 µg of D_3_R tagged to the ProLink fragment (ARMS2-PK2) either mono- or cotransfected with 0.5 µg of untagged NTSR1. The total amount of receptor was determined by saturation binding experiments to 240 fmol ∙ mg^-1^ protein (*K*_*D*_ 0.43 nM) for the D_3_R and 4,400 fmol ∙ mg^-1^ protein (*K*_D_ 1.4 nM) for the NTSR1. For NTSR1 monoexpression, 0.5 µg NTSR1 tagged with the ProLink fragment (PK1) and 2 µg Mock DNA were transfected and the expression determined to 3,100 fmol ∙ mg^-1^ protein (*K*_D_ 1.9 nM). 24 h after transfection, cells were detached using Versene (Invitrogen), resuspended in cell plating reagent 7 (DiscoverX) and seeded in white 384-well plates with clear bottom, (Greiner Bio one) at a density of 5,000 cells per well. After cultivation at 37 °C, 5% CO_2_ for 24 h, cells were stimulated with the test compounds for 5 h at 37 °C after preincubation with 1 µM YM254890 for 5 min, 1 µM haloperidol for 30 min or without preincubation. 10 µL of the detection mix were added per well chemiluminescence was measured with a CLARIOstar microplate reader after incubation for 60 min in the dark at room temperature. Data were normalised to the basal luminescence and analysed by three-parameter sigmoid or bell-shaped nonlinear regression using the algorithms of GraphPad Prism 6.0.

### BRET internalisation

HEK293SL cells^71^ or Δβ-arrestin-HEK^49^ were detached with Trypsin/EDTA (Wisent Inc., St-Bruno, QC, Canada) and 350,000 cells were transiently transfected in suspension using polyethyleneimine. 5 ng of NTSR1-Rluc or 400 ng of D_3_R-Rluc8 were either mono-or cotransfected with 600 ng of untagged D_3_R or 5 ng NTSR1, respectively, along with 300 ng rGFP-CAAX or 300 ng rGFP-FYVE and ssDNA to a total of 1 µg DNA. Cells were seeded in white poly-L-ornithine (Sigma Aldrich) coated 96-well plates (Greiner Bio one) at a density of 35,000 cells per well. 48 hours after transfection, cells were washed once with PBS and serum starved for 30–45 min in Tyrode’s Buffer at 37 °C, 5% CO_2_. Coelenterazine-h was added in a final concentration of 3 µM and cells were further incubated at 37 °C, 5% CO_2_. After 3 min, ligands were added and cells were stimulated for 30 min, 37 °C, 5% CO_2_ before determining the BRET_ratio_ using a Mithras LB940 multimode microplate reader with 480-20 nm (donor) and 530-20 nm (acceptor) filters. For kinetic experiments, cells were incubated with 3 µM coelenterazine-h for 8 min at 37 °C, 5% CO_2_., before 1 µM quinpirole or NT(8-13) were added and BRET measurements were immediately started. Ligand-induced effects were monitored by calculation of ΔBRET, which was determined as the difference in BRET_ratio_ between the ligand-treated conditions and vehicle-treated controls. Data were analysed by three-parameter sigmoid or bell-shaped nonlinear regression using the algorithms provided by GraphPad Prism 6.0.

### Statistics and reproducibility

In general, data are presented as mean ± s.e.m. or mean and individual data points from (*n*) biologically independent experiments. For the individual experiments, (*n*) is indicated in the tables and figure legends. For BRET saturation curves, graphs show results from representative experiments, with the individual data points denoting technical replicates. Experiments were repeated at least three times with high reproducibility. Statistical analyses were performed using GraphPad Prism 6.0 and 8.4 for Windows. BRET saturation experiments were analysed using two-tailed, paired Student’s *t* test and *p* < 0.05 was considered as statistically significant (Supplementary Table 2).

### Reporting summary

Further information on research design is available in the Nature Research Reporting Summary linked to this article.

## Supplementary information

Supplementary Information

Description of Additional Supplementary Files

Supplementary Movie 1.

Supplementary Data 1

Reporting Summary

## Data Availability

The data that support the findings of this study are available within the Supplementary Information, source data for Figs. 2–4, 6 and 7 is available as Supplementary Data 1 and/or from the corresponding authors upon reasonable request.

## References

[1] Bouvier M (2001). Oligomerization of G-protein-coupled transmitter receptors. Nat. Rev. Neurosci..

[2] Han Y, Moreira IS, Urizar E, Weinstein H, Javitch JA (2009). Allosteric communication between protomers of dopamine class A GPCR dimers modulates activation. Nat. Chem. Biol..

[3] Jordan BA, Devi LA (1999). G-protein-coupled receptor heterodimerization modulates receptor function. Nature.

[4] Terrillon S, Barberis C, Bouvier M (2004). Heterodimerization of V1a and V2 vasopressin receptors determines the interaction with β-arrestin and their trafficking patterns. Proc. Natl Acad. Sci. USA.

[5] White JF (2007). Dimerization of the class A G protein-coupled neurotensin receptor NTS1 alters G protein interaction. Proc. Natl Acad. Sci. USA.

[6] Botta J, Appelhans J, McCormick PJ (2020). Continuing challenges in targeting oligomeric GPCR-based drugs. Prog. Mol. Biol. Transl. Sci..

[7] Hiller C, Kühhorn J, Gmeiner P (2013). Class A G-protein-coupled receptor (GPCR) dimers and bivalent ligands. J. Med. Chem..

[8] Daniels DJ, Kulkarni A, Xie Z, Bhushan RG, Portoghese PS (2005). A bivalent ligand (KDAN-18) containing δ-antagonist and κ-agonist pharmacophores bridges δ2 and κ1 opioid receptor phenotypes. J. Med. Chem..

[9] Shonberg J, Scammells PJ, Capuano B (2011). Design strategies for bivalent ligands targeting GPCRs. ChemMedChem.

[10] Waldhoer M (2005). A heterodimer-selective agonist shows in vivo relevance of G protein-coupled receptor dimers. Proc. Natl Acad. Sci. USA.

[11] Hübner H (2016). Structure-guided development of heterodimer-selective GPCR ligands. Nat. Commun..

[12] Sokoloff P, Giros B, Martres MP, Bouthenet ML, Schwartz JC (1990). Molecular-cloning and characterization of a novel dopamine receptor (D3) as a target for neuroleptics. Nature.

[13] Joyce JN, Millan MJ (2005). Dopamine D3 receptor antagonists as therapeutic agents. Drug Discov. Today.

[14] Yang P, Perlmutter JS, Benzinger TLS, Morris JC, Xu J (2020). Dopamine D3 receptor: A neglected participant in Parkinson disease pathogenesis and treatment?. Ageing Res. Rev..

[15] Montoya A (2019). Dopamine receptor D3 signalling in astrocytes promotes neuroinflammation. J. Neuroinflammation.

[16] Levant B (1998). Differential distribution of D3 dopamine receptors in the brains of several mammalian species. Brain Res..

[17] Levesque, D. et al. Identification, characterization, and localization of the dopamine D3 receptor in rat brain using 7-[^3^H]hydroxy-N,N-di-n-propyl-2-aminotetralin. *Proc. Natl Acad. Sci. USA* **89**, 8155–8159 (1992). 10.1073/pnas.89.17.8155 PMC49875 1518841

[18] Ikemoto S (2007). Dopamine reward circuitry: two projection systems from the ventral midbrain to the nucleus accumbens–olfactory tubercle complex. Brain Res. Rev..

[19] Le Foll B (2014). Dopamine D3 receptor ligands for drug addiction treatment: update on recent findings. Prog. Brain Res..

[20] Sokoloff P, Le Foll B (2017). The dopamine D3 receptor, a quarter century later. Eur. J. Neurosci..

[21] Heidbreder CA, Newman AH (2010). Current perspectives on selective dopamine D3 receptor antagonists as pharmacotherapeutics for addictions and related disorders. Ann. N. Y. Acad. Sci..

[22] Newman AH, Grundt P, Nader MA (2005). Dopamine D3 receptor partial agonists and antagonists as potential drug abuse therapeutic agents. J. Med. Chem..

[23] Sarrieau A (1985). Characterization and autoradiographic distribution of neurotensin binding sites in the human brain. Brain Res..

[24] Boudin H, Pélaprat D, Rostène W, Beaudet A (1996). Cellular distribution of neurotensin receptors in rat brain: immunohistochemical study using an antipeptide antibody against the cloned high affinity receptor. J. Comp. Neurol..

[25] Sarret, P. & Cavelier, F. *Neurotensin and Its Receptors in Reference Module in Neuroscience and Biobehavioral Psychology* (Elsevier, 2017).

[26] Dobbs LK, Morikawa H (2020). Biasing neurotensin receptor signaling to arrest psychostimulant abuse. Cell.

[27] Barak LS (2016). ML314: a biased neurotensin receptor ligand for methamphetamine abuse. ACS Chem. Biol..

[28] Slosky LM (2020). β-Arrestin-biased allosteric modulator of NTSR1 selectively attenuates addictive behaviors. Cell.

[29] Dijkman PM (2018). Dynamic tuneable G protein-coupled receptor monomer-dimer populations. Nat. Commun..

[30] Borroto-Escuela DO (2013). Dopamine D2 receptor signaling dynamics of dopamine D2-neurotensin 1 receptor heteromers. Biochem. Biophys. Res. Commun..

[31] Koschatzky S, Tschammer N, Gmeiner P (2011). Cross-receptor interactions between dopamine D2L and neurotensin NTS1 receptors modulate binding affinities of dopaminergics. ACS Chem. Neurosci..

[32] Perron A, Sharif N, Sarret P, Stroh T, Beaudet A (2007). NTS2 modulates the intracellular distribution and trafficking of NTS1 via heterodimerization. Biochem. Biophys. Res. Commun..

[33] Hwang JR (2010). Intermolecular cross-talk between NTR1 and NTR2 neurotensin receptor promotes intracellular sequestration and functional inhibition of NTR1 receptors. Biochem. Biophys. Res. Commun..

[34] Plach M (2019). Differential allosteric modulation within dopamine D2R—neurotensin NTS1R and D2R—serotonin 5-HT2AR receptor complexes gives bias to intracellular calcium signalling. Sci. Rep..

[35] Binder EB, Kinkead B, Owens MJ, Nemeroff CB (2001). Neurotensin and dopamine interactions. Pharmacol. Rev..

[36] Diaz J (1994). Opposing roles for dopamine D2 and D3 receptors on neurotensin mRNA expression in nucleus accumbens. Eur. J. Neurosci..

[37] Koschatzky S, Gmeiner P (2012). Selective agonists for dopamine/neurotensin receptor heterodimers. ChemMedChem.

[38] Liu Y, Hillefors-Berglund M, von Euler G (1994). Modulation of dopamine D3 receptor binding by N-ethylmaleimide and neurotensin. Brain Res..

[39] Fehler SK (2014). Fast and efficient ^18^F-labeling by [^18^F]fluorophenylazocarboxylic esters. Chem. Eur. J..

[40] Maschauer S (2014). In vivo monitoring of the antiangiogenic effect of neurotensin receptor-mediated radiotherapy by small-animal positron emission tomography: a pilot study. Pharmaceuticals.

[41] Stanwood GD, Lucki I, McGonigle P (2000). Differential regulation of dopamine D2 and D3 receptors by chronic drug treatments. J. Pharmacol. Exp. Ther..

[42] Betancur C (1998). Characterization of binding sites of a new neurotensin receptor antagonist, [^3^H]SR 142948A, in the rat brain. Eur. J. Pharmacol..

[43] Ullmann T (2021). Homobivalent dopamine D2 receptor ligands modulate the dynamic equilibrium of D2 monomers and homo- and heterodimers. ACS Chem. Biol..

[44] Gomes I, Sierra S, Devi LA (2016). Detection of receptor heteromerization using in situ proximity ligation assay. Curr. Protoc. Pharmacol..

[45] Hall MP (2012). Engineered luciferase reporter from a deep sea shrimp utilizing a novel imidazopyrazinone substrate. ACS Chem. Biol..

[46] Borroto-Escuela DO, Flajolet M, Agnati LF, Greengard P, Fuxe K (2013). Bioluminescence resonance energy transfer methods to study G protein-coupled receptor-receptor tyrosine kinase heteroreceptor complexes. Methods Cell Biol..

[47] Marcellino D (2008). Identification of dopamine D1–D3 receptor heteromers: indications for a role of synergistic D1–D3 receptor interactions in the striatum. J. Biol. Chem..

[48] Peng Q, Shen J (2019). YM-254890 is a general inhibitor of G proteins. FASEB J..

[49] Cahill TJ (2017). Distinct conformations of GPCR–β-arrestin complexes mediate desensitization, signaling, and endocytosis. Proc. Natl Acad. Sci. USA.

[50] Besserer-Offroy É (2017). The signaling signature of the neurotensin type 1 receptor with endogenous ligands. Eur. J. Pharmacol..

[51] Kobayashi H, Picard L-P, Schönegge A-M, Bouvier M (2019). Bioluminescence resonance energy transfer–based imaging of protein–protein interactions in living cells. Nat. Protoc..

[52] Kim K-M (2001). Differential regulation of the dopamine D2and D3 receptors by G protein-coupled receptor kinases and β-arrestins. J. Biol. Chem..

[53] Kim K-M, Gainetdinov RR, Laporte SA, Caron MG, Barak LS (2005). G Protein-coupled receptor kinase regulates dopamine D3 receptor signaling by modulating the stability of a receptor-filamin-β-arrestin complex: a case of autoreceptor regulation. J. Biol. Chem..

[54] Yin W (2019). A complex structure of arrestin-2 bound to a G protein-coupled receptor. Cell Res..

[55] Huang W (2020). Structure of the neurotensin receptor 1 in complex with β-arrestin 1. Nature.

[56] Namkung Y (2016). Monitoring G protein-coupled receptor and beta-arrestin trafficking in live cells using enhanced bystander BRET. Nat. Commun..

[57] Oakley RH, Laporte SA, Holt JA, Barak LS, Caron MG (2001). Molecular determinants underlying the formation of stable intracellular G protein-coupled receptor-β-arrestin complexes after receptor endocytosis. J. Biol. Chem..

[58] Heakal Y, Kester M (2009). Nanoliposomal short-chain ceramide inhibits agonist-dependent translocation of neurotensin receptor 1 to structured membrane microdomains in breast cancer cells. Mol. Cancer Res..

[59] Chun L, Zhang W-h, Liu J-f (2012). Structure and ligand recognition of class C GPCRs. Acta Pharmacol. Sin..

[60] Hern JA (2010). Formation and dissociation of M1 muscarinic receptor dimers seen by total internal reflection fluorescence imaging of single molecules. Proc. Natl Acad. Sci. USA.

[61] Tabor A (2016). Visualization and ligand-induced modulation of dopamine receptor dimerization at the single molecule level. Sci. Rep..

[62] Calebiro D (2013). Single-molecule analysis of fluorescently labeled G-protein–coupled receptors reveals complexes with distinct dynamics and organization. Proc. Natl Acad. Sci. USA.

[63] Möller J (2020). Single-molecule analysis reveals agonist-specific dimer formation of µ-opioid receptors. Nat. Chem. Biol..

[64] Cheng Z-J, Miller LJ (2001). Agonist-dependent dissociation of oligomeric complexes of G protein-coupled cholecystokinin receptors demonstrated in living cells using bioluminescence resonance energy transfer. J. Biol. Chem..

[65] Kasai RS, Ito SV, Awane RM, Fujiwara TK, Kusumi A (2018). The class-A GPCR dopamine D2 receptor forms transient dimers stabilized by agonists: detection by single-molecule tracking. Cell Biochem. Biophys..

[66] Busnelli M (2016). Design and characterization of superpotent bivalent ligands targeting oxytocin receptor dimers via a channel-like structure. J. Med. Chem..

[67] Hamdan FF, Percherancier Y, Breton B, Bouvier M (2006). Monitoring protein-protein interactions in living cells by bioluminescence resonance energy transfer (BRET). Curr. Protoc. Neurosci..

[68] Marsango S (2017). A molecular basis for selective antagonist destabilization of dopamine D3 receptor quaternary organization. Sci. Rep..

[69] Zhang X, Sun N, Zheng M, Kim K-M (2016). Clathrin-mediated endocytosis is responsible for the lysosomal degradation of dopamine D3 receptor. Biochem. Biophys. Res. Commun..

[70] Cho E-Y (2007). Roles of protein kinase C and actin-binding protein 280 in the regulation of intracellular trafficking of dopamine D3 receptor. Mol. Endocrinol..

[71] Robertson DN (2016). Design and construction of conformational biosensors to monitor ion channel activation: a prototype FlAsH/BRET-approach to Kir3 channels. Methods.

[72] Guo W (2008). Dopamine D2 receptors form higher order oligomers at physiological expression levels. EMBO J..

[73] Quoyer J (2013). Pepducin targeting the C-X-C chemokine receptor type 4 acts as a biased agonist favoring activation of the inhibitory G protein. Proc. Natl Acad. Sci. USA.

[74] Paradis JS (2015). Receptor sequestration in response to β-arrestin-2 phosphorylation by ERK1/2 governs steady-state levels of GPCR cell-surface expression. Proc. Natl Acad. Sci. USA.

[75] Guan XM, Kobilka TS, Kobilka BK (1992). Enhancement of membrane insertion and function in a type IIIb membrane protein following introduction of a cleavable signal peptide. J. Biol. Chem..

[76] Hübner H, Haubmann C, Utz W, Gmeiner P (2000). Conjugated enynes as nonaromatic catechol bioisosteres: synthesis, binding experiments and computational studies of novel dopamine receptor agonists recognizing preferentially the D3 subtype. J. Med. Chem..

[77] Lowry OH, Rosebrough NJ, Farr AL, Randall RJ (1951). Protein measurement with the folin phenol reagent. J. Biol. Chem..

[78] Basden AG, Haniff CA, Mackay CD (2003). Photon counting strategies with low-light-level CCDs. Mon. Not. R. Astron. Soc..

[79] Azzari L, Foi A (2016). Variance stabilization for noisy+estimate combination in iterative poisson denoising. IEEE Signal Process. Lett..

