# Supplementary material for: Bivalent ligands promote endosomal trafficking of the dopamine D3 receptor-neurotensin receptor 1 heterodimer
Source: Commun Biol. 2021 Sep 10;4:1062. doi: 10.1038/s42003-021-02574-4 (PMC8433439; doi:10.1038/s42003-021-02574-4)
Supplement: Supplementary file 1 — Supplementary Information [file 42003_2021_2574_MOESM1_ESM.pdf]

Supplementary Information for

Bivalent ligands promote endosomal trafficking of the dopamine D3 receptor-neurotensin receptor 1 heterodimer

Julian Budzinski[1], Simone Maschauer[2], Hiroyuki Kobayashi[3], Pierre Couvineau[3], Hannah Vogt[1], Peter Gmeiner[1], Anna Roggenhofer[1], Olaf Prante[2], Michel Bouvier[3,*], Dorothee Weikert[1,*]

[1] Department of Chemistry and Pharmacy, Medicinal Chemistry, Friedrich-Alexander-Universität Erlangen-Nürnberg, 91058 Erlangen, Germany

[2] Department of Nuclear Medicine, Molecular Imaging and Radiochemistry, Friedrich-Alexander-Universität Erlangen-Nürnberg, 91054 Erlangen, Germany

[3] Department of Biochemistry and Molecular Medicine, Institute for Research in Immunology and Cancer, Université de Montréal, Montreal, QC, Canada, H3T-1J4

* Correspondence should be addressed to:

Michel Bouvier, email: michel.bouvier@umontreal.ca
Dorothee Weikert, email: dorothee.weikert@fau.de

This PDF file includes:

- Supplementary Figures 1 to 12
- Supplementary Tables 1 to 3
- Supplementary Methods
- Supplementary References

Supplementary Figures 1-12

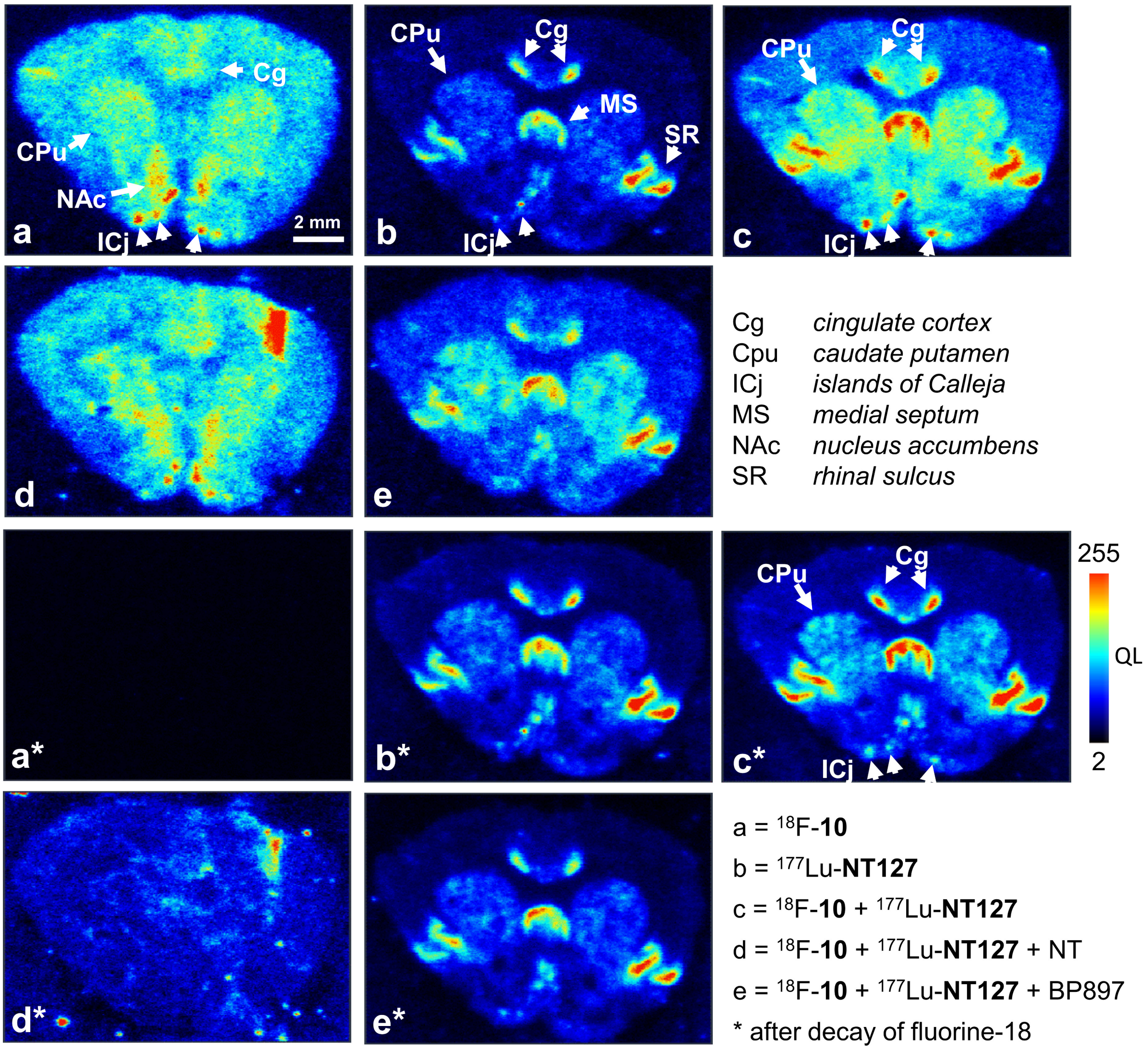

Supplementary Figure 1. *In vitro* rat brain autoradiography with the D$_3$R-selective radioligand ^{18}F-10 and the NTSR1-selective radioligand ^{177}Lu-NT127. Panels **a-e** show binding of ^{18}F-**10** alone (**a**), ^{177}Lu-**NT127** alone (**b**), simultaneous incubation of both radioligands (**c**), and in the presence of an excess of neurotensin (NT, 1 μM) (**d**) or the D$_3$R ligand BP897 (50 nM) (**e**). After complete radioactive decay (24 h) of ^{18}F, autoradiography was repeated to allow the detection of ^{177}Lu-**NT127** binding only (panels **a***-**e***). The intensity is shown as quantum level (QL) as provided by the software Quantity One.

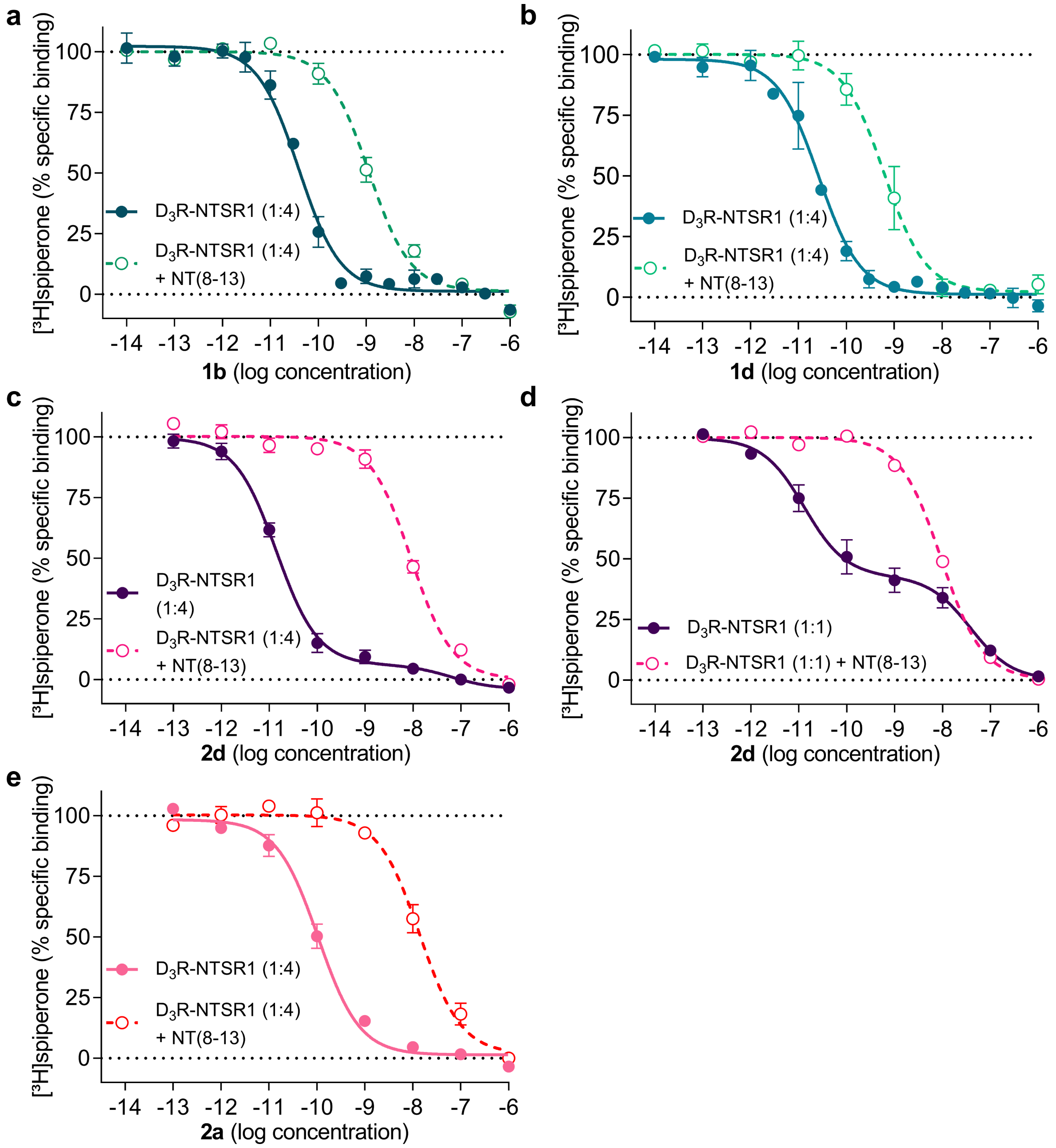

Supplementary Figure 2. Radioligand competition with membranes from D_3R-NTSR1-expressing cells and the radioligand [^{3}H]spiperone in presence and absence of 1 µM NT(8-13). (**a-e**) High affinity binding of ligands **1b**,**d** and **2a**,**d** is observed in membranes from HEK293T cells coexpressing D_3R and NTSR1. Displacement curves are predominantly monophasic if NTSR1 expression exceeds D_3R expression (relative ratio ~4:1). Coincubation with high concentrations (1 µM) of the monovalent NTSR1-ligand NT(8-13) leads to a significant reduction in binding affinity of the bivalent ligands **1b** (n = 10 (4 + NT(8-13)), **1d** (n = 5 (4 + NT(8-13)), **2a** (n = 5 (5 +NT(8-13)) and **2d** (n = 6 (3 + (NT(8-13)), as shown by the right-shift of the concentration-response curves. (**d**) If radioligand binding experiments are conducted with membranes from cells expressing equal amounts of both receptors (1:1 stoichiometry), biphasic binding curves are observed for the bivalent ligand **2d** (n = 3). In presence of 1 µM NT(8-13) (n = 2), the high-affinity component is depleted, leading to a monophasic displacement curve corresponding to a low-affinity bivalent ligand-receptor interaction. Data points represent mean ± s.e.m. of (n*)* independent experiments, each performed in triplicates.

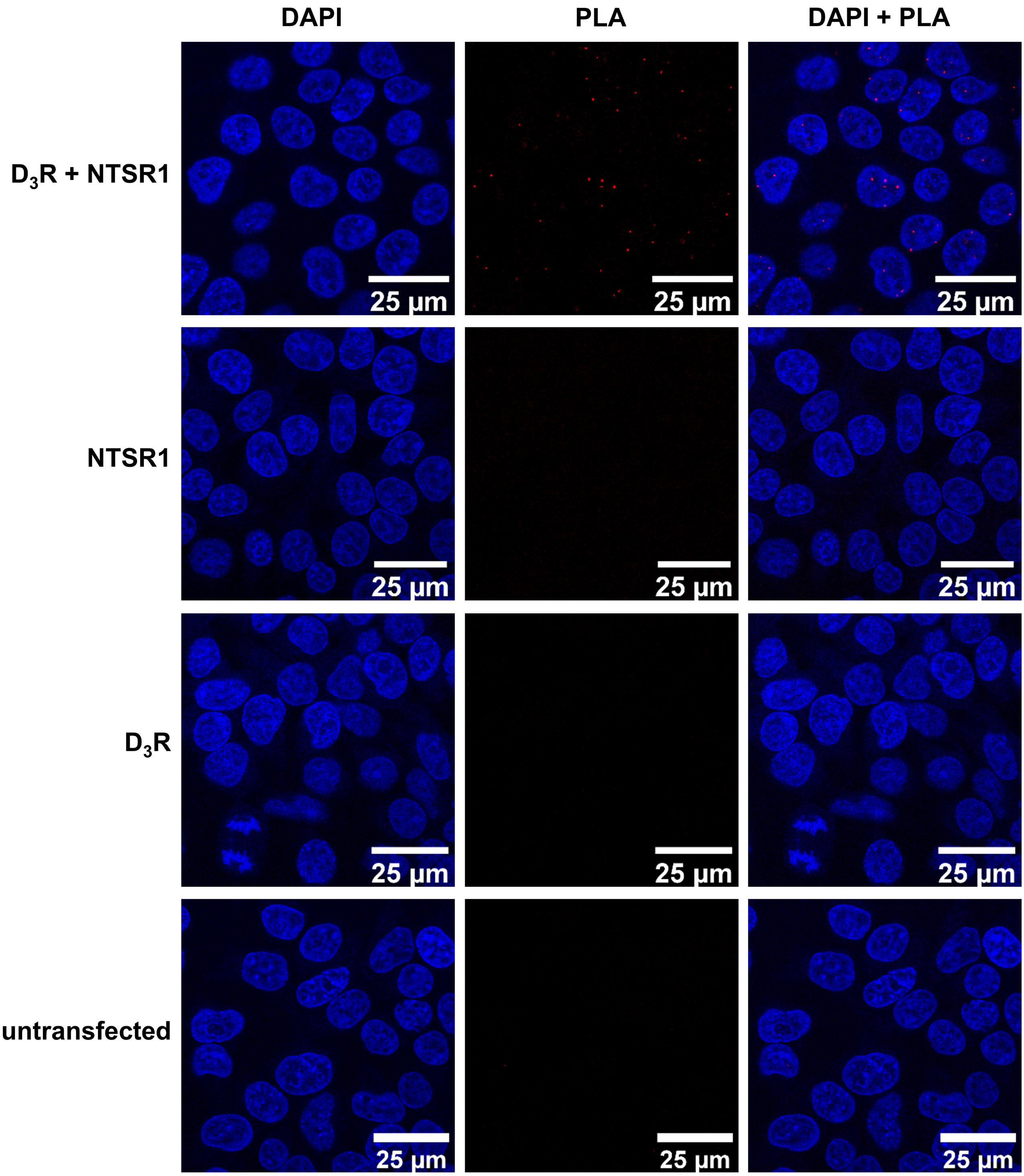

Supplementary Figure 3. ***In situ*** **proximity ligation assay (PLA) with D_3R and NTSR1 in HEK293T cells.** Direct *in situ* PLA was performed with anti-D_3R (ab42114, Abcam) conjugated to the PLUS-PLA probe and anti-NTSR1 (ab117592, Abcam) conjugated to the MINUS-PLA probe and imaged on a laser scanning confocal microscope at 63x magnification as described in Supplementary Methods. Only in cells expressing D_3R and NTSR1 red dots are observed, that correspond to the positive PLA signal, indicating close proximity (< 40 nm) of the two receptors.

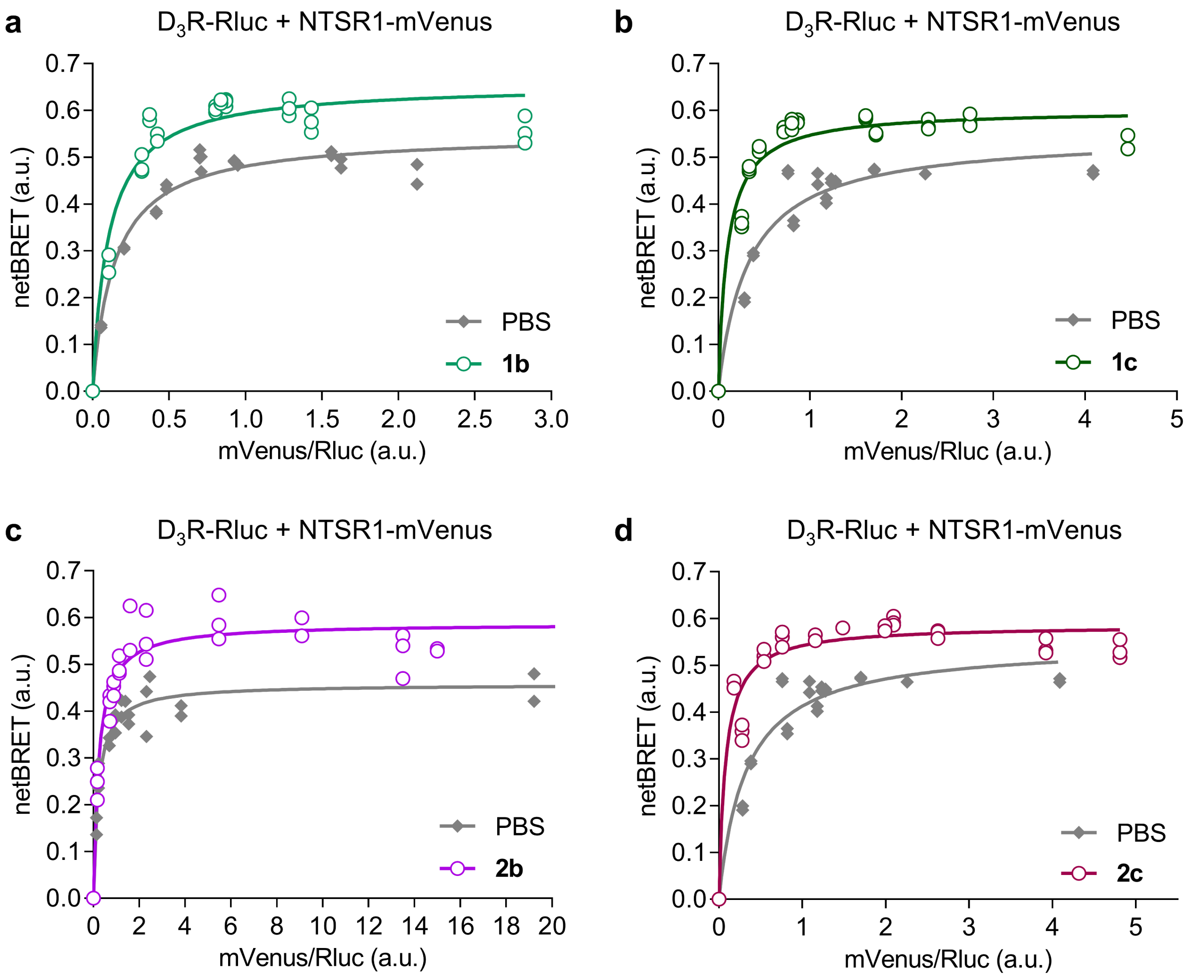

Supplementary Figure 4. BRET titration experiments with D$_3$R-Rluc-NTSR1-mVenus in presence of the bivalent ligands 1b,c and 2b,c. (**a-d**) BRET Saturation experiments with D$_3$R and NTSR1 in HEK293T cells in the presence (10 nM) and absence of bivalent ligands show that bivalent ligands foster the protein-protein interaction as indicated by the observed increase in BRET$_{max}$. Data show individual replicates for one representative out of at least three independent experiments carried out with each condition in triplicates (duplicates for PBS).

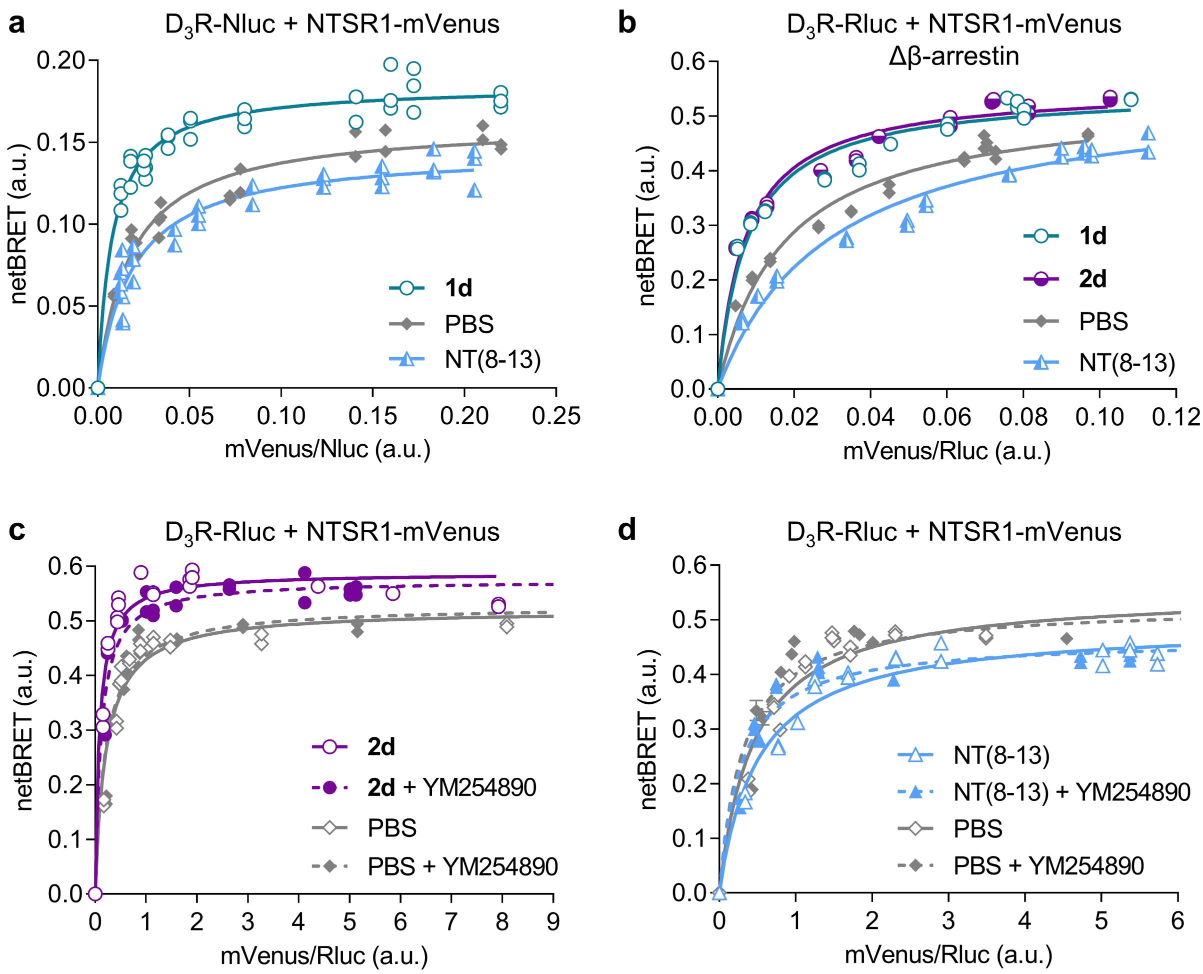

Supplementary Figure 5. BRET Saturation control experiments. (**a**) Replacement of Rluc by Nluc does not change the shape of the hyperbolic BRET saturation curve and the effects of ligands **1d** (10 nM) and NT(8-13) (1 µM). (**b**) BRET titration experiments with D_3R-Rluc and NTSR1-mVenus in HEK293 cells lacking β-arrestin1 and β-arrestin2 result in hyperbolic saturation curves in the absence of ligands. While incubation with bivalent compounds **1d** and **2d** (10 nM) increases $BRET_{max}$ and reduces $BRET_{50}$, incubation with 1 µM NT(8-13) decreases $BRET_{max}$ similar to wild-type HEK293 cells. (**c**,**d**) Inhibition of $G_{q/11}$ by YM254890 (1 µM, 5 min preincubation) has no influence on the hyperbolic shape of BRET saturation curves and does not impair the ability of compound **2d** and NT(8-13) to increase or decrease $BRET_{max}$, respectively. Data show individual replicates for one representative experiment carried out with each condition at least in duplicates.

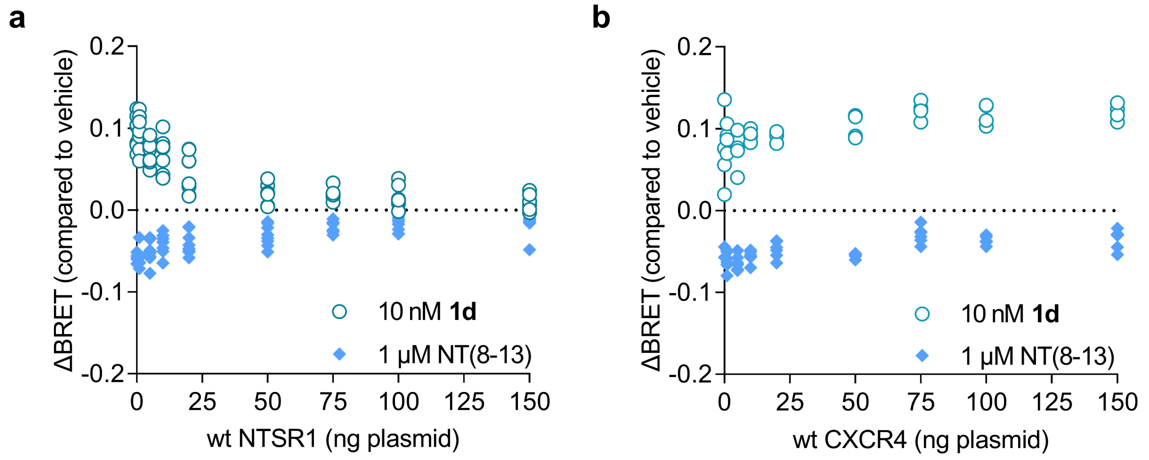

Supplementary Figure 6. BRET displacement experiments. Increasing amounts of wild-type (**a**) NTSR1 and (**b**) CXCR4 encoding plasmids were cotransfected to a constant amount of D_3R-Rluc (500 ng) and NTSR1-mVenus (100 ng) plasmids. The increase in $BRET_{max}$ elicited by 10 nM **1d** compared to vehicle treated cells (positive ΔBRET) is lost upon coexpression of wild-type NTSR1, and the decrease in BRETmax by 1 μM NT(8-13) is gradually diminished. Coexpression of CXCR4 does not decrease ΔBRET for **1d** and has only a minor effect for NT(8-13). Data show individual results from n = 8 independent experiments for NTSR1 and n = 4 independent experiments for CXCR4.

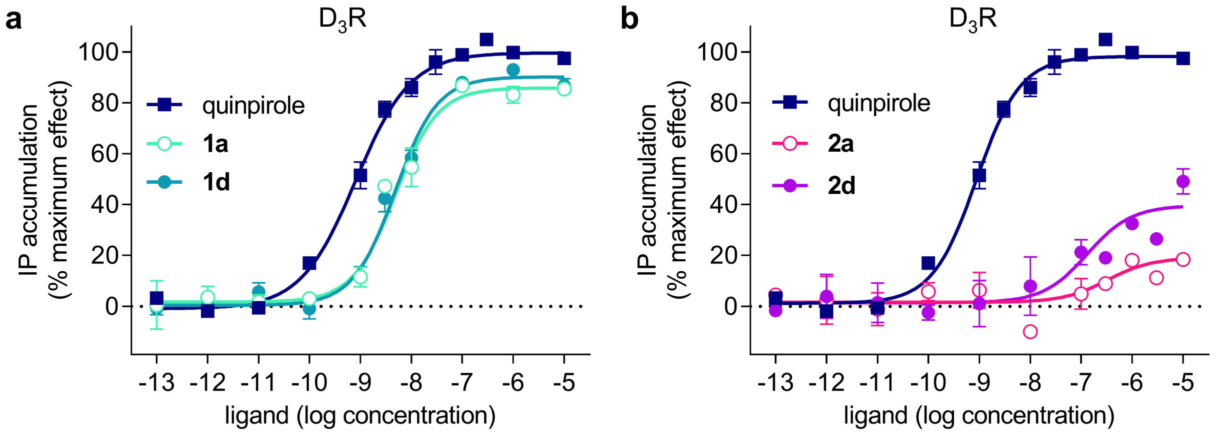

Supplementary Figure 7. Activation of D_3R in transiently transfected HEK293T cells. D_3R-mediated activation of G proteins was measured by stimulation of HEK293T cells transiently transfected with D_3R and a promiscuous G_q protein (G_{qprom}). Subsequent formation of IP_3 as second messenger of the G_q signaling pathway was determined employing the IPOne assay (CisBio). Compared to the reference agonist quinpirole (n = 9), (**a**) indanylamine-based ligands **1a** (n = 5) and **1d** (n = 5) are full D_3R agonists while (**b**) phenylpiperazine-based ligands **2a** (n = 3) and **2d** (n = 3) show weak partial agonist or antagonist-like behavior. Data show mean ± s.e.m. derived from (n) independent experiments.

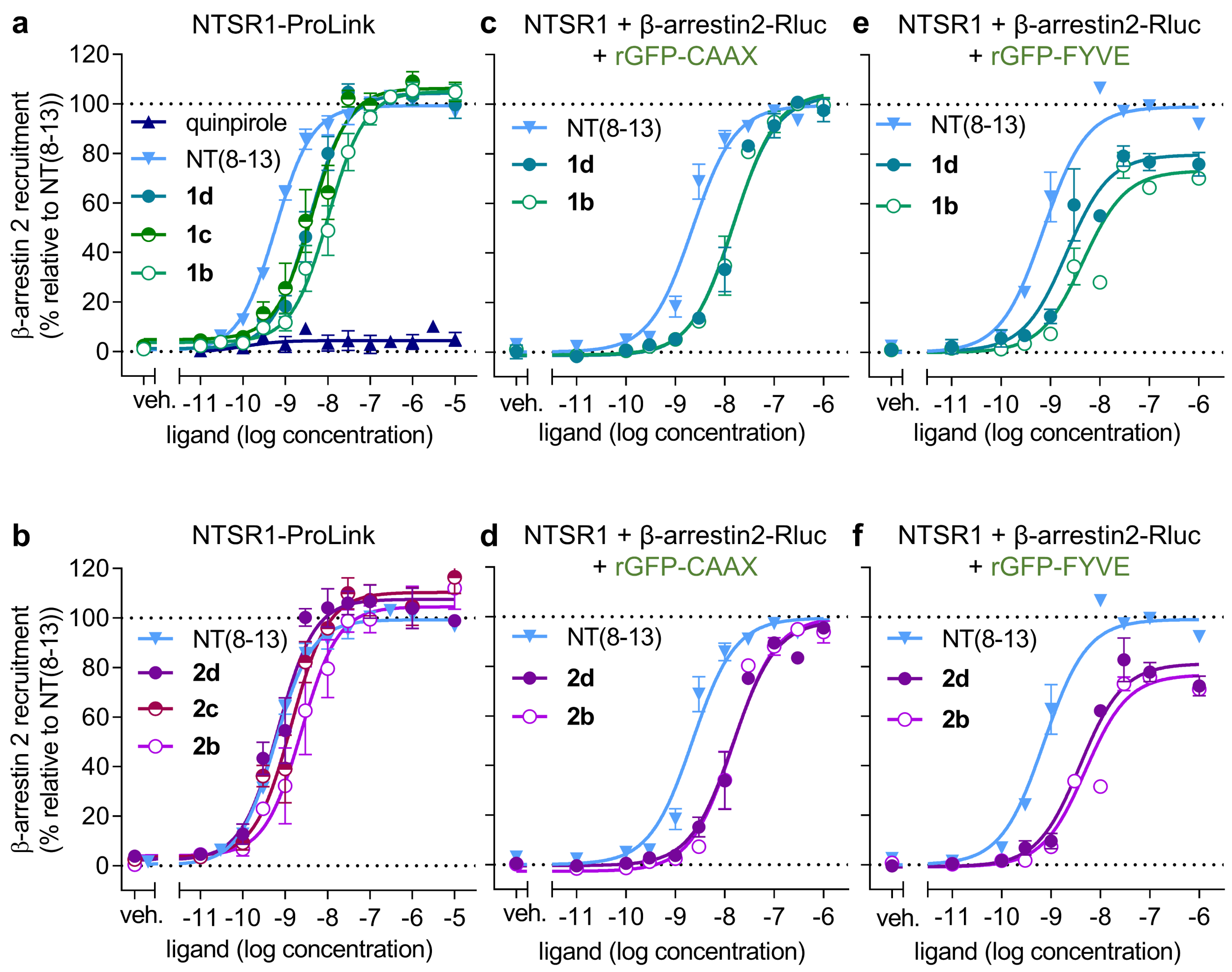

Supplementary Figure 8. β-arrestin2 recruitment to monoexpressed NTSR1 detected by enzyme complementation (DiscoverX) and bystander BRET. (**a**,**b**) NT(8-13) (n = 8) and bivalent ligands **1b-d** (n = 8 for **1b**,**c**; n = 7 for **1d**) and **2b-d** (n = 4) lead to a concentration-dependent recruitment of β-arrestin2, while quinpirole (n = 3) has no effect on NTSR1-ProLink activation. (**c**,**d**). Bivalent ligands (n = 3) and NT(8-13) (n = 4) induce recruitment of β-arrestin2-Rluc to the plasma membrane as detected by BRET with the plasma membrane marker rGFP-CAAX. (**e**,**f**) Compared to NT(8-13) (n = 3), the maximum effect (E_{max}) of the bivalent ligands (n = 3) is slightly lower when β-arrestin2 recruitment to early endosomes is investigated in HEK293 cells coexpressing β-arrestin2-Rluc, NTSR1 and rGFP-FYVE. All error bars denote s.e.m. derived from (n) independent experiments.

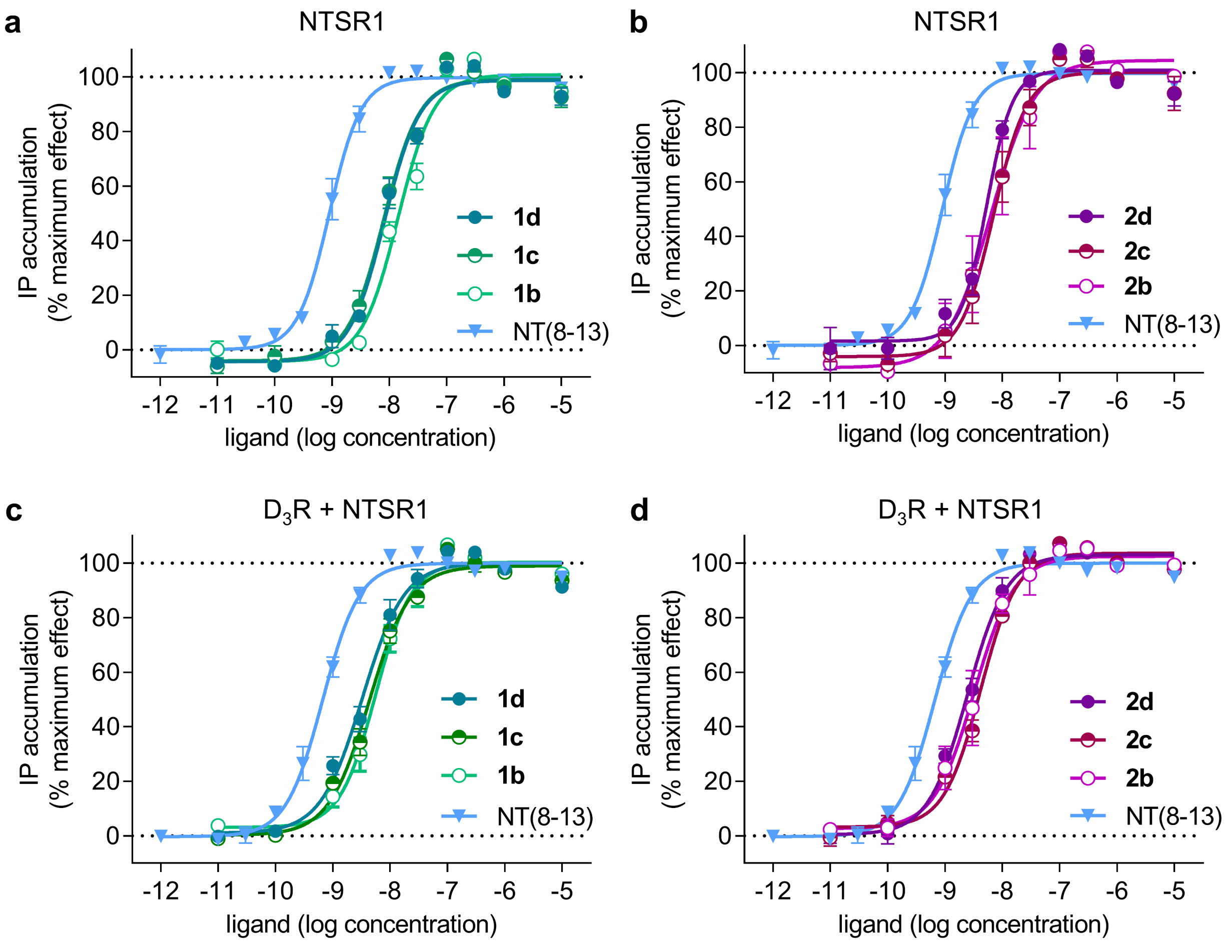

Supplementary Figure 9. Activation of NTSR1 in monoexpressing and D_3R-NTSR1 coexpressing HEK293T cells. NTSR1 activation of G_q proteins was investigated by measurement of the second messenger IP_3 employing the IPOne assay (CisBio). (**a**,**b**) Regardless of the D_3R pharmacophore, bivalent ligands (n = 4 for **1b**,**d** and n = 3 for **1c** and **2b-d**) activate monoexpressed NTSR1 with a slightly lower potency than NT(8-13) (n = 5). (**c**,**d**) Coexpression of the D_3R and the NTSR1 slightly decreases the EC_{50} of the bivalent compounds (n = 4 for **1b**,**d**; and n = 3 for **1c** and **2b-d**), while the EC_{50} of NT(8-13) (n = 7) remains unchanged compared to monoexpressed NTSR1. Error bars denote s.e.m. derived from (n) independent experiments.

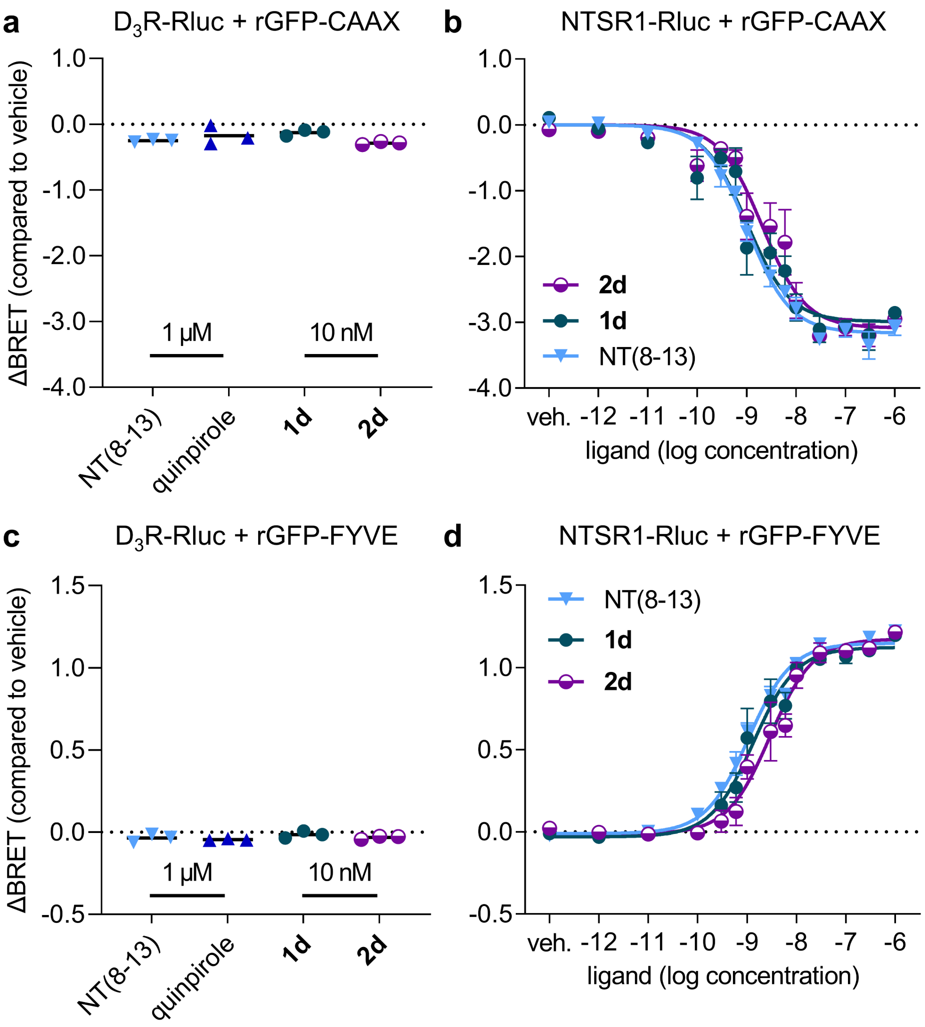

Supplementary Figure 10. Receptor internalization in D$_3$R or NTSR1 monoexpressing cells determined by bystander BRET. (**a**) No decrease in membrane BRET is observed in cells expressing D$_3$R-Rluc and rGFP-CAAX upon treatment with 1 µM quinpirole, 1 µM NT(8-13) or 10 nM bivalent ligands **1d** or **2d**. (**b**) Stimulation of HEK293SL cells expressing NTSR1-Rluc and rGFP-CAAX with NT(8-13) or the bivalent ligands **1d** or **2d** leads to a decrease of membrane BRET, indicative of NTSR1 sequestration/internalization (n = 4). (**c**) Stimulation of D$_3$R-Rluc expressing HEK293SL cells does not lead to receptor internalization to endosomes, independent of the ligand used. (**d**) Stimulation with NT(8-13) or the bivalent ligands **1d**, **2d** leads to a concentration-dependent trafficking of NTSR1 to endosomes, indicated by an increase in BRET between NTSR1-Rluc and the endosome marker rGFP-FYVE (n = 3). Data are displayed as (**a**,**c**) individual results and mean, (**b**,**d**) mean ± s.e.m from n biologically independent experiments.

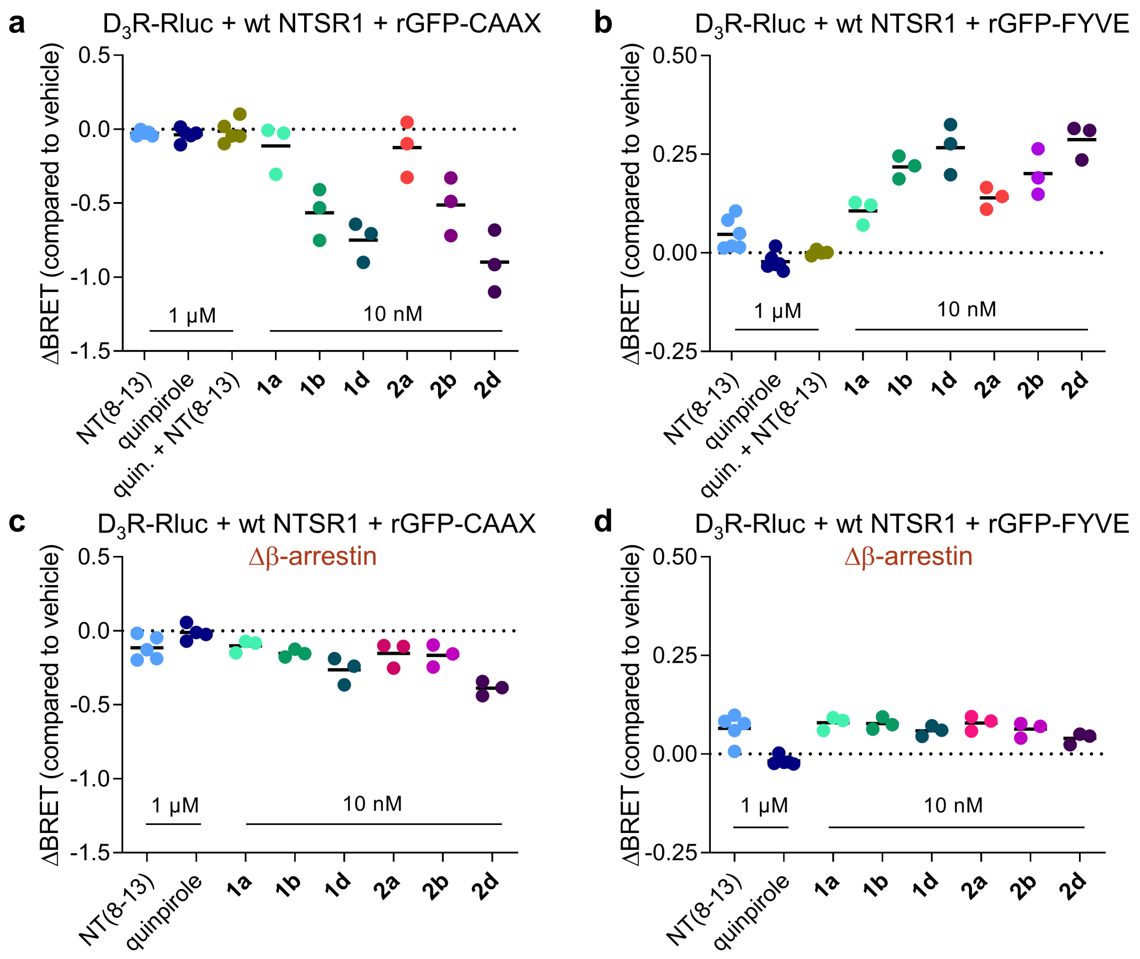

Supplementary Figure 11. Internalization in presence and absence of β-arrestin determined by bystander BRET in HEK293SL cells. (**a**,**b**) Stimulation of cells coexpressing D_3R-Rluc and wild-type NTSR1 with the bivalent ligands results in the D_3R trafficking from the membrane to endosomes. No cointernalization is detectable after stimulation with quinpirole, while NT(8-13) is able to induce a small increase in the endosomal compartment. (**c**,**d**) The specific effect of the bivalent compounds is almost completely lost as D_3R-Rluc, wild-type NTSR1 and rGFP-CAAX or rGFP-FYVE are coexpressed in HEK cells lacking β-arrestins. While quinpirole has no effect on the BRET signal, a slight decrease and increase, respectively, can be observed for NT(8-13) and the bivalent ligands. ΔBRET was calculated as the difference in $BRET_{ratio}$ between the ligand-treated conditions and vehicle-treated controls. Data show mean and individual results of at least three biologically independent experiments.

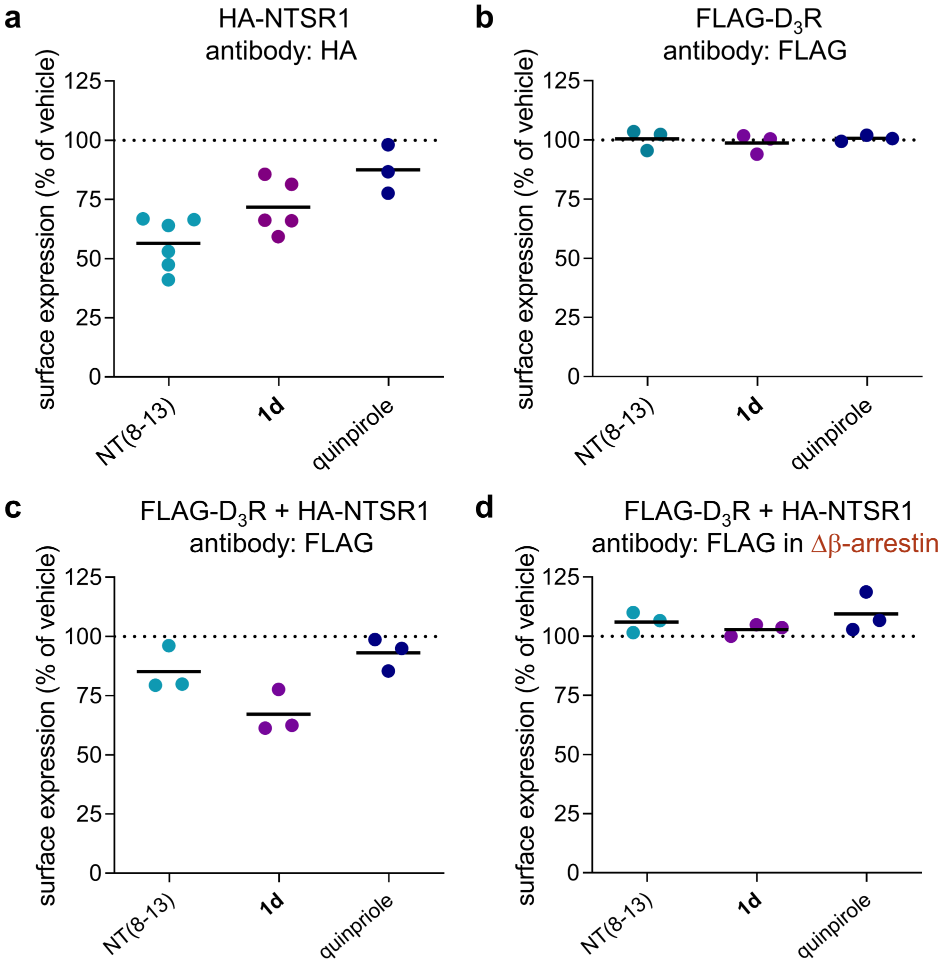

Supplementary Figure 12. Surface ELISA indicates internalization of D_3R only upon coexpression with NTSR1. Expression of D_3R N-terminally tagged with the FLAG-Epitope and NTSR1 N-terminally fused to an HA-tag allows detection of receptors on the cell surface. (**a**) Stimulation of monoexpressed NTSR1 with NT(8-13) (1 µM) or ligand **1d** (10 nM) reduces receptor surface expression by 44% and 28%, respectively. Quinpirole (1 µM) has no substantial effect on the NTSR1 surface expression. (**b**) After 30 min stimulation with quinpirole, NT(8-13) or compound **1d,** the surface expression of monoexpressed D_3R is unchanged. (**c**) ELISA anti-FLAG in D_3R-NTSR1 coexpressing HEK cells is used to quantify internalization of D_3R. After stimulation with 10 nM compound **1d**, the surface expression of D_3R is reduced to 67%. Monovalent NT(8-13) (1 µM) reduces the surface expression of D_3R to 85%. (**d**) In HEK cells coexpressing D_3R and NTSR1 but lacking β-arrestins neither NT(8-13) nor compound **1d** influence the surface expression of the D_3R. Data show mean and individual results of at least three biologically independent experiments.

Supplementary Tables 1-3

Supplementary Table 1. D_3R and NTSR1 binding affinities of the bivalent ligands 1a,b,c,d and 2a,c,d.

Affinities for D_3R were determined with the radioligand [³H]spiperone in membranes from HEK293T cells with or without coexpression of NTSR1. Affinities for monomeric NTSR1 are taken from *Hübner et al.*[1] and were determined with the radioligand [³H]neurotensin and membranes from CHO cells stably expressing NTSR1.

	K_i				dimer selectivity	
	D_3R[a] (pM)	D_3R-NTSR1[a] (pM)	D_3R-NTSR1[a] + 1 μM NT(8-13) (pM)	NTSR1[b] (pM)	K_i (D_3R) / K_i (D_3R-NTSR1)	K_i (NTSR1) / K_i (D_3R-NTSR1)
**1a**	370 ± 140 (*3*)	67 ± 2 (*4*)	n.t.[c]	1,500 ± 100 (*2*)	5.5	22
**1b**	280 ± 100 (*5*)	33 ± 24 (*10*)	400 ± 180 (*4*)	860 ± 790 (*4*)	8.5	26
**1c**	290 ± 40 (*3*)	10 ± 4.8 (*3*)	n.t.[c]	340 ± 290 (*4*)	29	34
**1d**	850 ± 380 (*4*)	6.1 ± 3.2 (*5*)	150 ± 58 (*3*)	570 ± 120 (*2*)	140	93
**2a**	4,400 ± 2,000 (*3*)	20 ± 2.9 (*3*)	6,000 ± 220 (*3*)	2,600 ± 1,700 (*2*)	220	130
**2c**	9,500 ± 1,200 (*3*)	15 ± 8.3 (*3*)	n.t.[c]	240 ± 20 (*2*)	630	16
**2d**	7,000 ± 3,300 (*3*)	4.8 ± 0.8 (*5*)	3,500 ± 780 (*3*)	830 ± 90 (*2*)	1,460	170

[a] Data indicate mean ± s.e.m. of (*n*) independent experiments.

[b] Data indicate mean ± s.d. of (*n*) independent experiments, taken from *Hübner et al.*[1].

[c] n.t. not tested.

Supplementary Table 2. $BRET_{50}$ and $BRET_{max}$ values of bivalent ligands 1a-d and 2a-d.

$BRET_{50}$ and $netBRET_{max}$ values were derived from BRET saturation experiments with D_3R-Rluc and NTSR1-mVenus in the absence and presence of 10 nM bivalent ligand.

	$BRET_{50}$ (mVenus/Rluc, a.u.) [a]			$netBRET_{max}$ (a.u.) [a,b]		
	non-stimulated	stimulated	$\Delta BRET_{50}$	non-stimulated	stimulated	$\Delta BRET_{max}$
**1a**	0.21 ± 0.11 (*4*)	0.07 ± 0.02 (*4*)	-0.14	0.51 ± 0.02 (*4*)	0.59 ± 0.01 (*4*)	+0.08 ** (p = 0.0008)
**1b**	0.13 ± 0.01 (3)	0.06 ± 0.03 (3)	-0.07	0.50 ± 0.04 (3)	0.59 ± 0.05 (*3*)	+0.09 * (p = 0.0132)
**1c**	0.19 ± 0.07 (*3*)	0.08 ± 0.01 (*3*)	-0.11	0.57 ± 0.03 (*3*)	0.65 ± 0.04 (*3*)	+0.08 * (p = 0.0205)
**1d**	0.13 ± 0.06 (*3*)	0.10 ± 0.04 (*3*)	-0.03	0.44 ± 0.03 (*3*)	0.57 ± 0.02 (*3*)	+0.13 * (p = 0.0301)
**2a**	0.19 ± 0.09 (*4*)	0.16 ± 0.07 (*4*)	-0.03	0.48 ± 0.03 (*4*)	0.58 ± 0.03 (*4*)	+0.10 * (p = 0.0220)
**2b**	0.21 ± 0.09 (*3*)	0.17 ± 0.07 (*3*)	-0.04	0.46 ± 0.01 (*3*)	0.56 ± 0.01 (*3*)	+0.10 * (p = 0.0208)
**2c**	0.19 ± 0.07 (*3*)	0.07 ± 0.01 (*3*)	-0.12	0.57 ± 0.03 (*3*)	0.63 ± 0.04 (*3*)	+0.06 * (p = 0.0364)
**2d**	0.22 ± 0.06 (*6*)	0.13 ± 0.04 (*6*)	-0.09	0.46 ± 0.01 (*6*)	0.58 ± 0.01 (*6*)	+0.12 ** (p = 0.0005)

[a] Data indicate mean ± s.e.m. of (*n*) independent experiments.

[b] Significant differences as determined by paired two-tailed Student's t test (* $p < 0.05$, ** $p < 0.01$).

Supplementary Table 3. NTSR1-mediated activation of Gα_q in presence and absence of D_3R. Activation of the Gα_q pathway was studied at the level of the second messenger inositol-phosphate employing the IPOne FRET-technology (Cisbio).

	Gα_q activation [a]			
	NTSR1		D_3R-NTSR1	
	EC_{50} (nM)	E_{max} (%) [b]	EC_{50} (nM)	E_{max} (%) [b]
NT(8-13)	0.89 ± 0.15 (*7*)	100 (7)	0.73 ± 0.10 (*7*)	100 (*7*)
**1b**	14 ± 1 (*4*)	100 ± 2 (*4*)	5.8 ± 1.0 (*4*)	100 ± 1 (*4*)
**1c**	8.1 ± 1.6 (*3*)	99 ± 3 (*3*)	4.5 ± 0.8 (*3*)	99 ± 1 (*3*)
**1d**	8.5 ± 1.1 (*4*)	98 ± 3 (*4*)	3.5 ± 0.7 (*4*)	100 ± 2 (*4*)
**2b**	7.7 ± 2.6 (*3*)	104 ± 1 (*3*)	3.6 ± 1.1 (*3*)	102 ± 1 (*3*)
**2c**	7.6 ± 1.9 (*3*)	100 ± 4 (*3*)	4.2 ± 0.4 (*3*)	104 ± 1 (*3*)
**2d**	5.3 ± 0.3 (*3*)	101 ± 1 (*3*)	2.5 ± 0.4 (*3*)	103 ± 1 (*3*)

[a] Data indicate mean ± s.e.m. of (*n*) independent experiments.

[b] Relative to the maximum effect of NT(8-13).

Supplementary Methods

In situ proximity ligation (PLA)

PLA probes were obtained by conjugation of the primary rabbit polyclonal anti-D_3R antibody (ab42114, Abcam) to a PLUS oligonucleotide (DUO92009, Sigma Aldrich) as previously described[2] and the primary rabbit polyclonal anti-NTSR1 antibody (ab117592, Abcam) to a MINUS oligonucleotide (DUO92010, Sigma Aldrich) following the manufacturer's instructions. HEK293T cells were transiently transfected with the plasmids encoding wild-type D_3R (2 µg), and/or NTSR1 (0.5 µg) per 10 cm culture dish, using Mirus TransIT-293 at a DNA:TransIT-293 ratio of 1:3. The total amount of DNA was complemented to 2.5 µg with a mock pcDNA3.1 plasmid if necessary. After 24 h at 37 °C and 5% CO_2, $4 \cdot 10^5$ cells · mL^{-1} were transferred to a sterile coverslip, which was pretreated with poly-D-lysine. Cells were kept for further 24 h at 37 °C and 5% CO_2, before they were washed twice with PBS, followed by fixation with 4% paraformaldehyde for 15 min at room temperature (rt). Cells were washed trice with PBS, treated with 10 mM glycine in PBS for 20 min (rt), followed by two wash steps and permeabilization with 0.1% Triton X-100 for 5 min (rt), and two more wash steps with PBS. *In situ* PLA was then performed using the Duolink PLA fluorescence kit (Sigma-Aldrich) according to manufacturer's protocol and as previously described.[3] In brief, cells were blocked with DuoLink *in situ* blocking solution (Sigma Aldrich) for 60 min at 37 °C before incubation with the primary antibodies (1:100) linked to the PLUS (anti-D_3R) or MINUS (anti-NTSR1) PLA probes, respectively, in antibody dilution buffer overnight at 4 °C. The cells were washed twice for 5 min with wash buffer A and incubated for 30 min at 37 °C with the ligation solution. After two further washing steps (wash buffer A, 5 min), the polymerase solution was added and the cells were incubated for 100 min at 37 °C. Finally, the cells were washed twice for 10 min with wash buffer B and once for 1 min with 0.01% wash buffer B in Milli-Q. The coverslips were mounted with DuoLink mounting medium containing DAPI, dried under protection from light at 4 °C and sealed using clear nail polish. For imaging, a Leica Stellaris 8 Broadband WLL & Tau-Sense laser scanning confocal microscope equipped with a 63x glycerol objective was used. Excitation of the DAPI signal was detected between 430 to 590 nm with a laser intensity of 2% at 405 nm. The PLA excitation was detected in the range of 620 to 750 nm with a laser intensity of 5% at 594 nm. Images were analyzed and figures were prepared using Fiji[4,5].

β-arrestin 2 recruitment (bystander BRET)[6]

HEK293SL cells were diluted to a density of 350,000 cells · mL^{-1} and transiently transfected using PEI with 5 ng NTSR1, 300 ng rGFP-CAAX or 300 ng rGFP-FYVE, 4 ng β-arrestin2-RlucII and ssDNA to a total DNA amount of 1 µg. 35,000 cells were plated in a 96-well plate coated with poly L-ornithine and cultivated for 48 h at 37°C, 5% CO_2. On the day of the experiment, the cell medium was aspirated and each well was washed once with PBS and serum starved for 30-45 min. A Deep Blue C dilution was added in a final concentration of 2.5 µM. Following a 5 min incubation at 37°C, 5% CO_2, the ligand dilution in Tyrode's buffer was added. After additional stimulation for 5 min at 37°C, 5% CO_2, $BRET^2$ was determined using a Mithras LB940 multimode microplate reader with 400-70 nm (donor) and 515-20 nm (acceptor) filters preheated to 37°C. Obtained $BRET_{ratios}$ were normalized to vehicle conditions (0%) and the maximum stimulation by NT(8-13) (100%) and analyzed by three-parameter non-linear regression employing the models implemented in GraphPad Prism 6.0.

ELISA

For surface ELISA, 350,000 HEK293SL or Δβ-arrestin-HEK cells were transfected in suspension with 1 µg DNA consisting of 400 ng Flag-D_3R, 5 ng HA-NTSR1 or both and ssDNA using PEI. 35,000 cells were plated in a 96-well plate coated with poly-L-ornithin. After 48 h at 37°C, 5% CO_2, the cell medium was replaced with 100 µL PBS containing 1 µM NT(8-13), 1 µM quinpirole or 10 nM **1d** and the cells were further incubated for 30 min at 37°C. After removal of PBS, cells were immediately fixed with 3% PFA for 10 min at room temperature. Cells were then washed three times followed by 1 h blocking with either 2% (anti-Flag) or 0.5% (anti-HA) BSA solution in PBS. 50 µL of anti-Flag-HRP (Sigma Aldrich) in 1:10,000 dilution or anti-HA-HRP (Roche) in 1:2,000 dilution in blocking solution was added and cells were incubated for 1 h under light exclusion. Following three washing steps, Sigmafast OPD (Sigma Aldrich) was prepared according to the manufacturer's protocol and 100 µL were added per well. Following 5-15 min incubation under light exclusion, the reaction was stopped by addition of 3 M HCl and absorption was read at 492 nm. Measured absorption values were background corrected (subtraction of the signal obtained with non-transfected cells) and data were normalized (%) relative to the surface expression of vehicle-treated cells.

IPOne accumulation

HEK293T cells were transiently transfected with 2 µg of D_3R and 0.5 µg NTSR1; or 2 µg mock pcDNA3.1 and 0.5 µg NTSR1 plasmids; or 1 µg of D_3R together with 2 µg of G_{qprom} plasmids using TransIT293 in a DNA to reagent ratio of 1 : 3. After 24h, cells were detached using Versene (Invitrogen) and 10,000 cells were seeded in black 384-well plates (Greiner Bio one, Frickenhausen, Germany) and additionally cultivated for 24 h at 37°C, 5% CO_2. After replacement of the medium with 10 µL stimulation buffer (CisBio, Codolet, France), 5 µL of the test compounds dissolved in stimulation buffer were added. After 90 min at 37°C, 5 µL of IP1 reagent mix and 5 µL of AB reagent mix in lysis buffer (CisBio) were added, followed by a 60 min incubation at room temperature under light exclusion. The FRET ratio was determined using a CLARIOstar microplate reader with a filter set of 620-10 nm and 665-10 nm. A sigmoid curve was fitted using the algorithms for three-parameter non-linear regression provided by GraphPad Prism 6.0 and the data were normalized to the effect elicited by NT(8-13) or quinpirole.

Supplementary References

1. Hübner, H. *et al.* Structure-guided development of heterodimer-selective GPCR ligands. *Nat. Commun.* **7**, 12298 (2016).
2. Reyes-Resina, I. *et al.* Expression of melatonin and dopamine D3 receptor heteromers in eye ciliary body epithelial cells and negative correlation with ocular hypertension. *Cells* **9**, 152 (2020).
3. Gomes, I., Sierra, S. & Devi, L. A. Detection of receptor heteromerization using in situ proximity ligation assay. *Curr. Protoc. Pharmacol.* **75**, 2.16.11-12.16.31 (2016).
4. Schindelin, J. *et al.* Fiji: an open-source platform for biological-image analysis. *Nat. Methods* **9**, 676-682 (2012).
5. Rueden, C. T. *et al.* ImageJ2: ImageJ for the next generation of scientific image data. *BMC Bioinf.* **18**, 529 (2017).
6. Namkung, Y. *et al.* Monitoring G protein-coupled receptor and beta-arrestin trafficking in live cells using enhanced bystander BRET. *Nat. Commun.* **7**, 12178 (2016).
