# Supplementary material for: Bivalent ligands promote endosomal trafficking of the dopamine D3 receptor-neurotensin receptor 1 heterodimer
Source: Commun Biol. 2021 Sep 10;4:1062. doi: 10.1038/s42003-021-02574-4 (PMC8433439; doi:10.1038/s42003-021-02574-4)
Supplement: Supplementary file 2 — Description of Additional Supplementary Files [file 42003_2021_2574_MOESM2_ESM.pdf]

Description of Additional Supplementary Files

File name: Supplementary Data 1.
Description: Source data for Figures 2-4, 6 and 7.

File name: Supplementary Movie 1.
Description: Time-lapse imaging of the effect of **1d** on the interaction between D_3R and NTSR1. HEK293SL cells were transfected with D3R-Nluc and NTSR1-mVenus. 10 µM coelenterazine 400a was added as a substrate. **1d** (10 nM) was added 4 min after starting continuous BRET image recording. The time-lapse is 300x speed (1 s in the movie corresponds to 300 s of measurement). BRET levels from 0.05 to 0.4 are expressed as a heat map color code from blue to red. Adjustments for the correction of the photon counting saturation and Poissonian denoising were applied to the entire images as described in the Methods section. The field of view of the movie is 137 µm x 137 µm.
