# Supplementary material for: Bivalent ligands promote endosomal trafficking of the dopamine D3 receptor-neurotensin receptor 1 heterodimer
Source: Commun Biol. 2021 Sep 10;4:1062. doi: 10.1038/s42003-021-02574-4 (PMC8433439; doi:10.1038/s42003-021-02574-4)
Supplement: Supplementary file 5 — Reporting Summary [file 42003_2021_2574_MOESM5_ESM.pdf]

nature research

Corresponding author(s): Michel Bouvier & Dorothée Weikert

Last updated by author(s): Jul 27, 2021

Reporting Summary

Nature Research wishes to improve the reproducibility of the work that we publish. This form provides structure for consistency and transparency in reporting. For further information on Nature Research policies, see our Editorial Policies and the Editorial Policy Checklist.

Statistics

For all statistical analyses, confirm that the following items are present in the figure legend, table legend, main text, or Methods section.

n/a	Confirmed	
☐	☒	The exact sample size (*n*) for each experimental group/condition, given as a discrete number and unit of measurement
☐	☒	A statement on whether measurements were taken from distinct samples or whether the same sample was measured repeatedly
☐	☒	The statistical test(s) used AND whether they are one- or two-sided<br>*Only common tests should be described solely by name; describe more complex techniques in the Methods section.*
☒	☐	A description of all covariates tested
☒	☐	A description of any assumptions or corrections, such as tests of normality and adjustment for multiple comparisons
☐	☒	A full description of the statistical parameters including central tendency (e.g. means) or other basic estimates (e.g. regression coefficient) AND variation (e.g. standard deviation) or associated estimates of uncertainty (e.g. confidence intervals)
☒	☐	For null hypothesis testing, the test statistic (e.g. *F*, *t*, *r*) with confidence intervals, effect sizes, degrees of freedom and *P* value noted<br>*Give P values as exact values whenever suitable.*
☒	☐	For Bayesian analysis, information on the choice of priors and Markov chain Monte Carlo settings
☒	☐	For hierarchical and complex designs, identification of the appropriate level for tests and full reporting of outcomes
☒	☐	Estimates of effect sizes (e.g. Cohen's *d*, Pearson's *r*), indicating how they were calculated

Our web collection on statistics for biologists contains articles on many of the points above.

Software and code

Policy information about availability of computer code

Data collection	Clario Star Software, version 5.40 (BMG Labtech); MikroWin 2000, version 4.18 (Berthold); MATLAB 2019b; Leica Application Suite X (LAS X).
Data analysis	Prism 6 for Windows, version 6.07 (GraphPad), Prism 8 for Windows, version 8.4.3 (GraphPad);<br>MARS, version 3.30 (BMG Labtech); Quantity One (BioRad); Microsoft Excel, Version 2016; MATLAB 2019b; ImageJ 1.52a.; Fiji.

For manuscripts utilizing custom algorithms or software that are central to the research but not yet described in published literature, software must be made available to editors and reviewers. We strongly encourage code deposition in a community repository (e.g. GitHub). See the Nature Research guidelines for submitting code & software for further information.

Data

Policy information about availability of data

All manuscripts must include a data availability statement. This statement should provide the following information, where applicable:

- Accession codes, unique identifiers, or web links for publicly available datasets
- A list of figures that have associated raw data
- A description of any restrictions on data availability

The data that support the findings of this study are available within the Supplementary Information, source data for Figures 2-4, 6 & 7 is available as Supplementary Data 1 and/or from the corresponding authors upon reasonable request.

Field-specific reporting

Please select the one below that is the best fit for your research. If you are not sure, read the appropriate sections before making your selection.

☒ Life sciences ☐ Behavioural & social sciences ☐ Ecological, evolutionary & environmental sciences

For a reference copy of the document with all sections, see nature.com/documents/nr-reporting-summary-flat.pdf

Life sciences study design

All studies must disclose on these points even when the disclosure is negative.

Sample size	Sample size was not predetermined.
Data exclusions	Data points were only excluded for technical reasons (e.g. pipetting errors).
Replication	All experiments have been performed in various (usually three or more) independent replication runs.
Randomization	Data generation and analysis was not randomized.
Blinding	Data analysis was not blinded.

Reporting for specific materials, systems and methods

We require information from authors about some types of materials, experimental systems and methods used in many studies. Here, indicate whether each material, system or method listed is relevant to your study. If you are not sure if a list item applies to your research, read the appropriate section before selecting a response.

Materials & experimental systems

n/a	Involved in the study	
☐	☒	Antibodies
☐	☒	Eukaryotic cell lines
☒	☐	Palaeontology and archaeology
☐	☒	Animals and other organisms
☒	☐	Human research participants
☒	☐	Clinical data
☒	☐	Dual use research of concern

Methods

n/a	Involved in the study	
☒	☐	ChIP-seq
☒	☐	Flow cytometry
☒	☐	MRI-based neuroimaging

Antibodies

Antibodies used	Mouse Monoclonal anti-FLAG M2-Peroxidase (HRP) (Sigma-Aldrich, M2-A8592)<br>Rat Monoclonal anti-HA-Peroxidase, High Affinity (3F10) (Roche, 12013819001)<br>Rabbit polyclonal anti-D3R antibody (Abcam, ab42114)<br>Rabbit polyclonal anti-NTSR1 antibody (Abcam, ab117592)
Validation	Anti-Flag and anti-HA antibodies were not further validated. Rabbit polyclonal anti-D3R and anti NTSR1 antibodies were tested by PLA against non-transfected HEK293T cells and showed no signals.

Eukaryotic cell lines

Policy information about cell lines

Cell line source(s)	HEK293SL and HEK b-Arrestin KO were gifted from Stephane Laporte (McGill University). HEK293T (ATCC accession number CRL-11268) were gifted from the Chair of Physiology (FAU Erlangen). HEK293 stably expressing b-arrestin fused to the enzyme acceptor were purchased from DiscoverX.
Authentication	Cell lines were not further authenticated.
Mycoplasma contamination	All cell lines were regularly confirmed to be free of mycoplasma contamination employing the PCR Mycoplasma Detection Kit (abm, G238) or Luminescence-based MycoAlert Plus Kit (Lonza, LT07-418).
Commonly misidentified lines<br>(See ICLAC register)	n.a.

Animals and other organisms

Policy information about studies involving animals; ARRIVE guidelines recommended for reporting animal research

Laboratory animals	Species: rat (Charles River); strain: Crl:CD(SD) / outbred; sex: female; age:22-23 weeks; one single animal was used for conducting in vitro rat brain autoradiography.
Wild animals	n.a.
Field-collected samples	n.a.
Ethics oversight	The animal experiments were approved by the local animal protection authorities (Government of Central Franconia, Germany, No. 55.2 2532-2-618-14) and performed at the FAU Erlangen-Nuernberg in accordance with the relevant institutional guidelines and EU regulations.

Note that full information on the approval of the study protocol must also be provided in the manuscript.
